# Seasonal dynamics of marine protist communities in tidally mixed coastal waters

**DOI:** 10.1111/mec.16539

**Published:** 2022-06-16

**Authors:** Mariarita Caracciolo, Fabienne Rigaut‐Jalabert, Sarah Romac, Frédéric Mahé, Samuel Forsans, Jean‐Philippe Gac, Laure Arsenieff, Maxime Manno, Samuel Chaffron, Thierry Cariou, Mark Hoebeke, Yann Bozec, Eric Goberville, Florence Le Gall, Loïc Guilloux, Anne‐Claire Baudoux, Colomban de Vargas, Fabrice Not, Eric Thiébaut, Nicolas Henry, Nathalie Simon

**Affiliations:** ^1^ CNRS Station Biologique de Roscoff AD2M, UMR7144 Sorbonne Université Roscoff France; ^2^ CNRS Station Biologique de Roscoff FR2424 Sorbonne Université Roscoff France; ^3^ UMR BGPI, F‐34398 Cirad Montpellier France; ^4^ Faculty of Biology Technion—Israel Institute of Technology Haifa Israel; ^5^ Research Federation for the study of Global Ocean Systems Ecology and Evolution FR2022/Tara Oceans GOSEE Paris France; ^6^ Laboratoire des Sciences du Numérique de Nantes (LS2N) CNRS, UMR6004 Ecole Centrale de Nantes Université de Nantes Nantes France; ^7^ Institut de recherche pour le développement (IRD) Délégation Régionale Ouest IMAGO Plouzané France; ^8^ CNRS, FR 2424, ABiMS Platform Station Biologique de Roscoff Sorbonne Université Roscoff France; ^9^ CNRS, IRD, CP53 Unité biologie des organismes et écosystèmes aquatiques (BOREA) Muséum National D’Histoire Naturelle Sorbonne Université Université de Caen Normandie Université des Antilles Paris France; ^10^ Mediterranean Institute of Oceanography (MIO) Marseille cedex 9 France; ^11^ CNRS, OSU STAMAR, UMS2017 Sorbonne Université Paris cedex 05 France

**Keywords:** annual succession, DNA metabarcoding, marine protists, temporal variability, time‐series data, Western English Channel

## Abstract

Major seasonal community reorganizations and associated biomass variations are landmarks of plankton ecology. However, the processes of plankton community turnover rates have not been fully elucidated so far. Here, we analyse patterns of planktonic protist community succession in temperate latitudes, based on quantitative taxonomic data from both microscopy counts (cells >10 μm) and ribosomal DNA metabarcoding (size fraction >3 μm, 18S rRNA gene) from plankton samples collected bimonthly over 8 years (2009–2016) at the SOMLIT‐Astan station (Roscoff, Western English Channel). Based on morphology, diatoms were clearly the dominating group all year round and over the study period. Metabarcoding uncovered a wider diversity spectrum and revealed the prevalence of Dinophyceae and diatoms but also of Cryptophyta, Chlorophyta, Cercozoa, Syndiniales and Ciliophora in terms of read counts and or richness. The use of morphological and molecular analyses in combination allowed improving the taxonomic resolution and to identify the sequence of the dominant species and OTUs (18S V4 rDNA‐derived taxa) that drive annual plankton successions. We detected that some of these dominant OTUs were benthic as a result of the intense tidal mixing typical of the French coasts in the English Channel. Our analysis of the temporal structure of community changes point to a strong seasonality and resilience. The temporal structure of environmental variables (especially Photosynthetic Active Radiation, temperature and macronutrients) and temporal structures generated by species life cycles and or species interactions, are key drivers of the observed cyclic annual plankton turnover.

## 1 INTRODUCTION

Annual succession of species ‐ and associated variations in biomass ‐ are one of the classical hallmarks of plankton ecology in both marine and freshwater systems (Margalef, 1978; Sommer et al., 1986, 2012; Winder and Cloern, 2010). In temperate biomes, annual plankton biomass patterns classically involve some regularity in form of a phytoplankton spring bloom that follows the increase of light availability in relation to a decrease in vertical mixing and nutrient availability, and provides food to grazers (Sverdrup, 1953; Cushing, 1959; Margalef, 1978). The resulting spring peak of zooplankton leads to the decline of phytoplankton towards a mid‐season biomass minimum while subsequent food limitation and fish predation controls zooplankton biomass (Sommer et al., 2012). The sequence of planktonic taxa emerging along the course of this rhythmic phenomenon depends on regional, ecological and biogeochemical specificities (e.g., coastal vs shelf vs oceanic conditions), but in a given habitat annually reoccurring species successions are commonly observed (see for example Egge et al., 2015; Marquardt et al., 2016; Modigh, 2001; Piredda et al., 2017; Ribera d'Alcalà et al., 2004). Annual succession of species have been described by early planktonologists (Allen, 1936; Gran & Braarud, 1935) and have inspired the founding theories of ecological successions (Margalef, 1963, 1958, 1978). In most regions, these seasonal cycles linked to plankton species phenology have probably governed the evolution of life cycles and migratory behaviours of organisms ranging from the smallest fishes to whales and birds (Cushing, 1959, 1990; Longhurst, 1998).

Identifying these temporal patterns and determining their principal environmental drivers are essential to reveal the mechanisms driving species succession and shaping community composition, and to predict how they will be modified by climate change (Edwards & Richardson, 2003; Siano et al., 2021). Decades of research have emphasized the major role of physical factors (e.g., light, temperature and turbulence) (Margalef, 1978; Townsend et al., 1992, 1994; Sommer et al., 2012; Barton et al., 2014) in pacing the annual oscillations of plankton biomass and diversity (Margalef, 1978; Townsend et al., 1992, 1994; Sommer et al., 2012; Barton et al., 2014). These factors, contingent to the annual climate cycle and operating across various astronomic and geological time scales, would control the dynamics of phytoplankton biomass (Cloern, 1996; Smetacek, 1985; Sommer et al., 1986) in a similar way to terrestrial plants (Craine et al., 2012; Richardson et al., 2010). However, seasonal successions are also an emergent property of the community dynamics, and the complex network of biotic interactions (e.g., predation, competition, parasitism, mutualism) could be the major force shaping annual plankton successions (Dakos et al., 2009; Drake, 1990; Logares et al., 2018). In other words, intrinsic biological factors, including functional traits (Edwards et al., 2013) or interactions within and between species and functional groups, could drive the dynamics and ensure the stability of marine plankton through time. The idea that biodiversity buffers ecosystem changes against environmental variations (Tilman, 1999; Tilman et al., 2006; Loreau & de Manzancourt, 2013) matches results obtained from manipulated microbiomes (Fernandez‐Gonzalez et al., 2016) and theoretical studies (Dakos et al., 2009).

Characterized by particularly high dispersal, large population sizes, and short generation time (Villarino et al., 2018), marine microorganisms represent about half of the overall carbon biomass and play key roles in global biogeochemical fluxes (Bar‐On & Milo, 2019; Falkowski et al., 2008). They are responsible for nearly all the primary production and respiration occurring in the marine realm (Moran, 2015). Annual species successions have been hard to demonstrate for microorganisms (i.e., viruses, bacteria, archaea and protists), especially for those with sizes under 10 μm which are difficult to identify under a microscope. However, the use of high throughput sequencing (HTS) and metagenomics approaches has shown that marine microbial communities exhibit clear annual patterns of species or operational taxonomic units (OTU) successions (Fuhrman et al., 2006, 2015; Gilbert et al., 2012; Bunse & Pinhassi, 2017; Giner et al., 2019; Käse et al., 2020). Given the extraordinary roles of microscopic plankton in ocean ecology, being able to document their dynamics in space and time is of tremendous importance to predict future changes that will occur in the next decades.

Here, we report pluri‐annual patterns of protists community dynamics off the French coast of the Western English Channel (WEC; Tréguer et al., 2014). The English Channel (EC) is an epicontinental sea which stands as a biogeographical crossroad between the warm‐temperate Atlantic system and the cold‐temperate North Sea and Baltic continental system of Northern Europe. It is a zone of high turbulence due to strong tidal currents. A seasonal thermocline occurring from May to October is only reported in its western entrance, offshore and along the UK coasts (Pingree & Griffiths, 1978, 1980). There are indications that current anthropogenic climate changes have already impacted pelagic and benthic compartments and affected the productivity of this shelf sea (see e.g., Beaugrand et al., 2002; Genner et al., 2004; Hiscock et al., 2004; Southward et al., 2005). Significant biological shifts, including species replacements or changes in species abundances and distributions, have been documented in the English Channel since over a century in response to climate change and other anthropogenic drivers (Boalch, 1987; Southward et al., 2005; Molinero et al., 2013; Mieszkowska et al., 2014; Reygondeau et al., 2015). Changes in the planktonic community composition have notably been observed at the L4 time‐series station by the Plymouth Western Channel Observatory (Barton et al., 2020; Edwards et al., 2013; Molinero et al., 2013; Pingree & Griffiths, 1978; Reygondeau et al., 2015; Widdicombe et al., 2010). The temporal dynamics of planktonic communities in the permanently well‐mixed waters that characterize the French coasts of the WEC have been less intensively studied.

In this study, plankton samples were collected over a period of 8 years (2009–2016) at the Roscoff time‐series station. We analysed protist cell counts (size fraction >10 μm) and protist of size fraction >3 μm with 18S V4 rDNA metabarcoding. Our aim was to (i) describe the seasonal dynamics of the protist communities, and (ii) explore how environmental factors (e.g., temperature, light, salinity, pH, and macronutrients) influence the dominant protist compartment over different time scales.

## 2 MATERIALS AND METHODS

### 2.1 Sampling location

**FIGURE 1:**
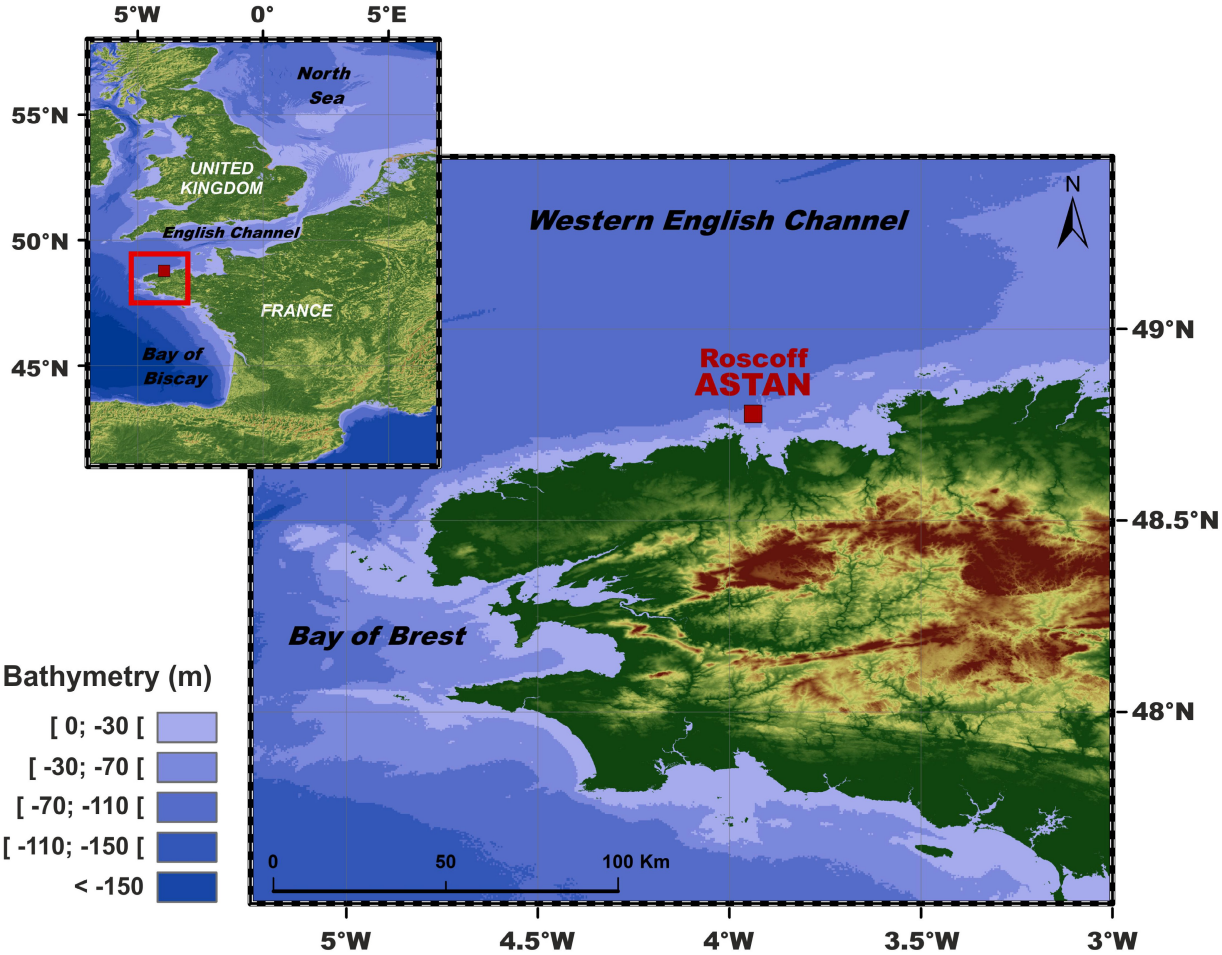
Location of the study area. The SOMLIT‐Astan sampling station (48:46′49″ N; 3:58′14″ W) is located in the Western English Channel, 3.5 km from the coast. The water column at this site is 60 m deep and is never stratified due to intense tidal mixing. The site is strongly impacted by storms in winter

The SOMLIT‐Astan sampling station is located in the western English Channel, 3.5 km off Roscoff (Brittany, France) (60 m depth, 48°46′18″ N–3°58′6″ W, Figure 1). Monitoring of the hydrology and phytoplankton at the SOMLIT‐Astan station has been implemented in 2000 (Guilloux et al., 2013), and is currently operated in the frame of the SOMLIT (Service d'Observation en Milieu LITtoral, since 2000, http://somlit.epoc.u‐bordeaux1.fr/) and PHYTOBS (PHYtoplankton OBServatory, since 2018) national monitoring programs. Hydrological and plankton samples are collected bimonthly aboard the research vessel Néomysis during high neap tide at surface (1 m depth) using a 5 L Niskin bottle. For this study, the data corresponding to the period 2009 to 2016 were analysed.

### 2.2 Environmental data

Meteorological data (rainfall height, wind speed and direction, global radiation) and hydrological data (temperature, nutrient concentrations, chlorophyll‐*a* biomass, particulate organic carbon and nitrogen, and suspended matter) for the period 2009 to 2016 were obtained from MétéoFrance (https://meteofrance.com/) and the SOMLIT program (https://www.somlit.fr/parametres‐et‐protocoles/), respectively. Mean daily tidal amplitude values were calculated from the water hourly heights available from the Service Hydrographique et Océanographique de la Marine (SHOM, https://data.shom.fr/), and used as a proxy of tidal mixing. Photosynthetically available radiations (PAR) and the diffuse attenuation coefficient for downwelling irradiance at 490 nm (Kd490) were obtained from the National Aeronautic and Space Administration (NASA, https://modis.gsfc.nasa.gov/data/dataprod/) and the National Oceanic and Atmospheric Administration (NOAA, https://coastwatch.pfeg.noaa.gov/). The average light received during the 8 days that preceded each sampling dates was calculated from PAR (PAR8days, extracted from https://modis.gsfc.nasa.gov/data/dataprod/par.php). Kd490, which is dependent on the availability of ratios of remote sensing reflectance (Rrs) in the blue‐green spectral region (e.g., 490–565 nm) was extracted from https://modis.gsfc.nasa.gov/data/dataprod/kd_490.php. The North Atlantic Oscillation index (NAO, Hurrell, 1995; Trigo et al., 2002) that influences the local meteorological conditions, was obtained from NOAA (https://www.ncdc.noaa.gov/teleconnections/nao/). Protocols used for the hydrological parameters by the SOMLIT are summarized below (Gac et al., 2020). Seawater temperature (T°C) was measured in situ using a Sea‐bird SBE19+ CTD profiler with an initial accuracy of ± 0.005°C. Discrete salinity samples were measured on a portal salinometer with a precision of 0.002. Nutrient concentrations (${{\text{NO}}^{-}}_{3}$, ${{\text{NO}}^{-}}_{2}$, ${{\text{PO}}_{4}}^{3-}$ and SiOH_4_) were determined using an AA3 auto‐analyser (Seal Analytical) following the method of Aminot and Kérouel (2007) with an accuracy of 0.02 μmol/L, 1 nmol/L, 1 nmol/L and 0.01 μmol/L for ${{\text{NO}}_{3}}^{-}$, ${{\text{NO}}_{2}}^{-}$, ${{\text{PO}}_{4}}^{3-}$ and SiOH_4_, respectively. Ammonium ($\text{NH}₄{}^{+}$) concentrations were determined using the indophenol blue method of Koroleff (1969). To determine chlorophyll‐*a* concentrations (Chl*‐a*), 0.5 L of seawater were filtered onto glass‐fibre filters (Whatman GF/F) and immediately frozen. Samples were extracted in 5 ml of acetone, acidified with HCl and Chl*‐a* concentrations, and were measured using a fluorometer (model 10 analogue fluorometer Turner Designs), with an estimated accuracy of 0.05 μg/L. Protocols used to measure the biomass of particulate organic carbon (POC), particulate organic nitrate (PON), suspended matter (MES) and the ratio of the two stable isotopes of nitrogen (DeltaN15) are described in the SOMLIT website. All parameters and their origins are resumed in Table 2.

### 2.3 Microscopic phytoplankton counts

Samples (250 ml) of natural seawater used for the acquisition of microscopic counts were preserved with acid Lugol's iodine (Sournia, 1978; Guilloux et al., 2013), stored in the dark, and further processed between 15 days and up to 1 year after sampling. Lugol's iodine was added either back in the laboratory 1.5 to 2 h after sampling or onboard immediately after sampling. Cell counts were obtained from subsamples that were gently poured into 50 ml composite settling chamber (HYDRO‐BIOS, Kiel), according to the standard Utermöhl settlement method (Sournia, 1978; Guilloux et al., 2013). For some winter samples characterized by lower cell numbers, 100 ml settlement chambers were used. Counts and identification of taxa were performed under an inverted light microscope (Leica DMI 300) at 200× and 400× magnification. References used for species identification included Tomas (1997), Throndsen et al. (2007), Hartley et al. (1996), Kraberg et al. (2010), Hoppenrath et al. (2009), Horner (2002) and the Plankton Net Data Provider (http://www.planktonnet.eu/). Taxonomic assignation was determined at lowest possible rank (e.g., species). Raw microscopic counts were regularly stored in a local MS‐Access database and uploaded in the RESOMAR PELAGOS (http://abims.sb‐roscoff.fr/pelagos/) national database. The morphological taxa contingency table was carefully examined to detect inconsistencies (e.g., abrupt changes in cell counts over the time series), and taxa for which identification was uncertain were grouped into broader taxonomic categories (i.e., *Fragilaria*/*Brockmaniella* or *Cylindrotheca closterium*/*Nitzschia longissima*). The final morphological data set (https://doi.org/10.5281/zenodo.5033180) (Rigaut‐Jalabert et al., 2021) consisted of counts of 146 taxonomical entities (taxa larger than 10 μm in size) across 185 dates from 2009 to 2016.

### 2.4 Protists DNA metabarcoding (size fraction >3 μm)

For the generation of DNA metabarcoding data, natural seawater from the Niskin bottle was transported to the laboratory in a 10 L Nalgene bottle and a volume of 5 L was collected onto 3 μm polycarbonate membranes (47 mm, Whatman). Filters were preserved in 1.5 ml of lysis buffer (sucrose 256 g/L, Tris 50 mM pH 8, EDTA 40 mM) and stored at −80°C until further processing. A total of 185 samples were collected between 2009 and 2016.

#### 2.4.1 DNA extraction, PCR amplification, and sequencing

Samples were first incubated 45 min at 37°C with 100 μl lysozyme (20 mg/ml), and 1 h at 56°C with 20 μl proteinase K (20 mg/ml) and 100 μl SDS 20%. Nucleic acids were then extracted using a phenol‐chloroform method (Sambrook et al., 1989), and purified using silica membranes from the NucleoSpin PlantII kit (Macherey‐Nagel). DNA was eluted with 100 μl Tris‐EDTA 1× pH 8 buffer and quantified using a Nanodrop ND‐1000 spectrophotometer and a Qubit 2.0 Fluorometer instrument with dsDNA high sensitivity (HS) assay (ThermoFisher Scientific). Total DNA extracts were then used as templates for PCR amplification of the V4 region of the 18S rRNA gene (∼380 bp) using the primers TAReuk454FWD1 (CCAGCASCYGCGGTAATTCC, *S. cerevisiae* position 565–584) and TAReukREV3 (ACTTTCGTTCTTGATYRA, *S. cerevisiae* position 964–981) (Stoeck et al., 2010) including Illumina adapters. These primers are known to target most eukaryotic groups (McNichol et al., 2021), although they do not perfectly match with sequences of haptophytes (Balzano et al., 2015). PCR reactions (25 μl) contained 1× Master Mix Phusion High‐Fidelity DNAPolymerase (Finnzymes; ThermoFisher), 0.35 μM of each primer, 3% dimethylsulphoxide and 5 ng of DNA. Each DNA sample was amplified in triplicates. The PCR program had an initial denaturation step at 98°C during 30 s, 10 cycles of denaturation at 98°C, annealing at 53°C for 30 s and elongation at 72°C for 30 s, then 15 similar cycles but with 48°C annealing temperature, and a final step at 72°C for 10 min. Polymerase chain reaction triplicates were pooled, purified and eluted (30 μl) with NucleoSpin Gel and PCR Clean‐Up kit (Macherey‐Nagel, ref: 740770.50 and 740770.250), and quantified with the Quant‐It PicoGreen double stranded DNA Assay kit (ThermoFisher). About 1 μg of pooled amplicons were sent to Fasteris (www.fasteris.com, Plan‐les‐Ouates, Switzerland) for high throughput sequencing on a 2 × 250 bp MiSeq Illumina. Sequences were obtained in five separate runs. Overall, ~7 million unique sequences were obtained for a total of 185 samples collected over the 8 years (>3 μm).

#### 2.4.2 Reads quality filtering and clustering

Generation of 18S V4 rDNA operational taxonomic units (OTUs) from the raw sequencing reads (deposited at the European Nucleotide Archive [ENA] under the project id PRJEB48571) and their assembly into a contingency table was obtained according to the following pipeline (https://doi.org/10.5281/zenodo.5791089). The paired‐end fastq files were demultiplexed and PCR primers were trimmed using Cutadapt v2.8 (Martin‐Jézéquel et al., 1992). Reads shorter than 100 nucleotides or untrimmed were filtered out. Trimmed paired‐end reads were merged using the fastq mergepairs command from VSEARCH v2.9.1 (Rognes et al., 2016) with a minimum overlap of 10 base pairs. Merged reads longer than 200 nucleotides were retained and clustered into OTUs using Swarm v2.2.2 with *d* = 1 and the fastidious option (Mahé et al., 2014, 2015). The most abundant sequence of each OTU is defined as the representative sequence. OTUs with a representative sequence considered to be chimeric by the uchime_denovo command from VSEARCH or with a quality per base below 0.0002 were filtered out. Finally, OTUs which appeared in less than two samples or with less than 3 reads were discarded (de Vargas et al., 2015).

#### 2.4.3 Taxonomic assignations

The V4 region was extracted from the 18S rDNA reference sequences from PR2 v4.12 (Guilloux et al., 2013) with Cutadapt, using the same primer pair as for the PCR amplification (maximum error rate of 0.2 and minimum overlap of 2/3 the length of the primer). The representative sequences of each OTU were compared to these V4 reference sequences by pairwise global alignment (usearch_global VSEARCH's command). Each OTU inherits the taxonomy of the best hit or the last common ancestor in case of ties. OTUs with a score below 80% similarity were considered as unassigned (Mahé et al., 2017; Stoeck et al., 2010). In this study, focusing on the ecology of protists, only OTUs assigned to protist lineages (eukaryotes which are not Metazoa, Rhodophyta, Phaeophyceae, Ulvophyceae or Streptophyta) were considered. The final data set (filtered OTU table, available at https://doi.org/10.5281/zenodo.5032450) (Henry et al., 2021) contained 185 samples with a total of ~12.7 million sequence reads and 15,271 OTUs affiliated to protist taxa. Because our approach is sensitive to the presence of wrong reference sequences, the taxonomy of the dominant OTUs (e.g., based on abundance and occurrence) was checked and refined manually by BLASTing them (https://blast.ncbi.nlm.nih.gov/Blast.cgi) against the nucleotide collection (nt). The origin and assignations of the best blast sequences (most of which were 100% similar to our sequences) and of the corresponding strains or isolates were carefully examined before taking the final taxonomic assignation decision (Table S1). Justifications of the taxonomic and phylogenetic assignation of the corresponding strains were systematically searched in the references cited along with accessions or in culture collections where applicable.

### 2.5 Statistical analysis

All statistical analyses were performed using R (R version 4.1.0, R Development Core Team, 2011). The R package “vegan” (Oksanen et al., 2013) and “data.table” (Dowle and Srinivasan, 2019) were used to analyse frequency count data, diversity, and to compute variance partitioning. The dbMEM analyses were performed using the packages “ade4” (Dray & Dufour, 2007), “adespatial” (Dray et al., 2018), “ape 5.0” (Paradis & Schliep, 2019) and “spdep” (Bivand & Wong, 2018). All figures were made with “ggplot2” (Wickham, 2016) (Figure S1, https://gitlab.com/MariaritaCaracciolo/roscoff‐astan‐time‐series). Unlike for cell counts, relative read abundance is considered for the metabarcoding analysis; samples were rarefied at 10.000 reads.

#### 2.5.1 Alpha and beta diversity

Standard alpha diversity metrics (Shannon Diversity Index and species richness) and beta diversity metrics (Jaccard similarity index and Bray–Curtis similarity index; Krebs, 1999; Legendre & Legendre, 1998) were calculated for both the morphological and metabarcoding data sets in order to analyse temporal changes in the composition and structure of the protist communities. Random subsampling (rarefaction) was used for the metabarcoding data set prior to the calculation of alpha diversity metrics (i.e., species richness and Shannon diversity index) and for the calculation of the Jaccard similarity index in order to account for differences in sequencing depth (i.e., total number of reads generated for a sample). Hellinger transformed data (Legendre & Gallagher, 2001) were used for the calculation of Bray‐Curtis dissimilarities.

#### 2.5.2 Temporal structure of protist communities

In order to detect the temporal structure of the communities, we used distance‐based Moran's eigenvector maps (dbMEM) (Legendre & Gauthier, 2014). This method has the potential to detect temporal structures produced by the species assemblage itself (through auto‐assemblage processes or autogenetic succession that involve species interactions, Connell and Slatyer (1977), Reynolds (1984) McCook (1994)) provided that all influential variables have been included in the analysis (Legendre & Gauthier, 2014). The dbMEM eigenfunctions were computed from a distance matrix of the time separating observations, truncated at a threshold corresponding to the largest time interval (lag = 44 days) (Legendre & Gauthier, 2014). A forward selection procedure implemented in the package adespatial (“forward.sel” function; Dray et al., 2018) was used to identify significant dbMEM. This analysis consists in a series of regressions performed on community matrices, that is, OTU read abundance (*n* = 15,271) or species cell counts (*n* = 146) data. Only OTUs present in at least 10 out of the 185 total samples were retained and the data were Hellinger‐transformed in order to (i) avoid overweighting rare species and (ii) be able to use Euclidean distances that allow to compute RDA (Legendre & Gallagher, 2001). Significant linear trends were then removed by computing the residuals, and ANOVA‐like tests (with 999 permutations; Legendre et al., 2011) were implemented on the RDA to assess the significance of each constrained axis (*p*‐value < .05). To calculate the proportion of the variance explained by the significant axes, the adjusted R^2^ of the RDA result was used. Variance partitioning analyses allowed to filter out the variations due to temporal structures, or autocorrelation, which accommodate the use of statistical tests to further assess which environmental variables can influence community dynamics and species composition. All parameters were first tested for collinearity, then successively used in a forward selection to identify those significant to be tested for the study. To interpret temporal variations, we calculated Spearman's rank correlation coefficients between the environmental parameters and the eigenvalues of the first three axes of the RDA.

## 3 RESULTS

### 3.1 Seasonal dynamics at the SOMLIT‐Astan station

**FIGURE 2:**
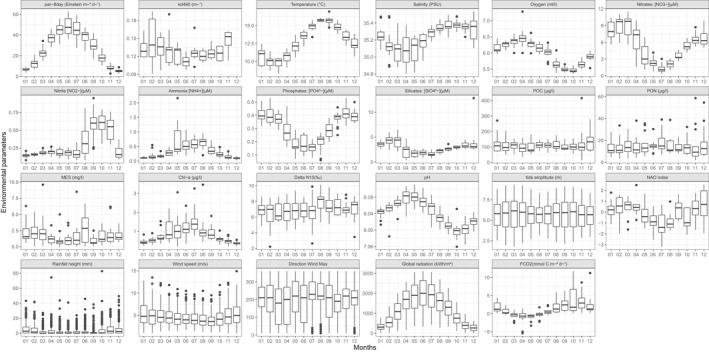
Monthly variations of the hydrological and meteorological parameters at the SOMLIT‐Astan station in the period 2009–2016. Sampling was carried out at high neap tides. PAR is the photosynthetically available radiation calculated as the average light received during the 8 days before sampling. Kd490 is intended as the diffuse attenuation coefficient for downwelling irradiance at 490 nm. Interannual variations of all parameters presented in this figure can be found in Figure S2

At the SOMLIT‐Astan time‐series station (Figure 1), both the hydrological parameters and phytoplankton biomass displayed clear seasonal patterns over the 8‐year period (2009–2016, Figure 2). In this tidally mixed environment, mean monthly temperatures varied from 9.8 (in March) to 15.7°C (in August). Mean monthly salinity ranged between 35.1 and 35.4 (from spring to autumn). Peaks of chlorophyll‐*a* (Chl‐*a)* biomass were generally recorded throughout summer (from June to August, Figure S2). From 2009 to 2016, mean monthly Chl‐*a* values were recorded between 0.4 and 1.5 μg/L (in December and July, respectively), and seasonal variations were synchronous with PAR (5.3 to 48.1 E m^−2^ day^−1^). Mean monthly minima in the main macronutrient concentration (${{\text{PO}}_{4}}^{3}$, ${{\text{SiO}}_{4}}^{2-}$ and ${{\text{NO}}_{2}}^{-}$) that sustain phytoplankton production were recorded in summer, when phytoplankton biomass was high; however, macronutrients were never completely depleted (Figure 2). Annual oscillations of pH were also recorded with minima in autumn. Although sampling occurred consistently during high neap tides, a clear biannual rhythm was detected in the mean monthly tidal amplitudes, which varied between 3.1 and 4.2 m with the highest mean values in late spring (May) according to the yearly change in the obliquity of the Earth's Equator. From 2009 to 2016, all parameters exhibited large inter‐annual variations and no significant decadal trend was detected (Figure S2).

**FIGURE 3:**
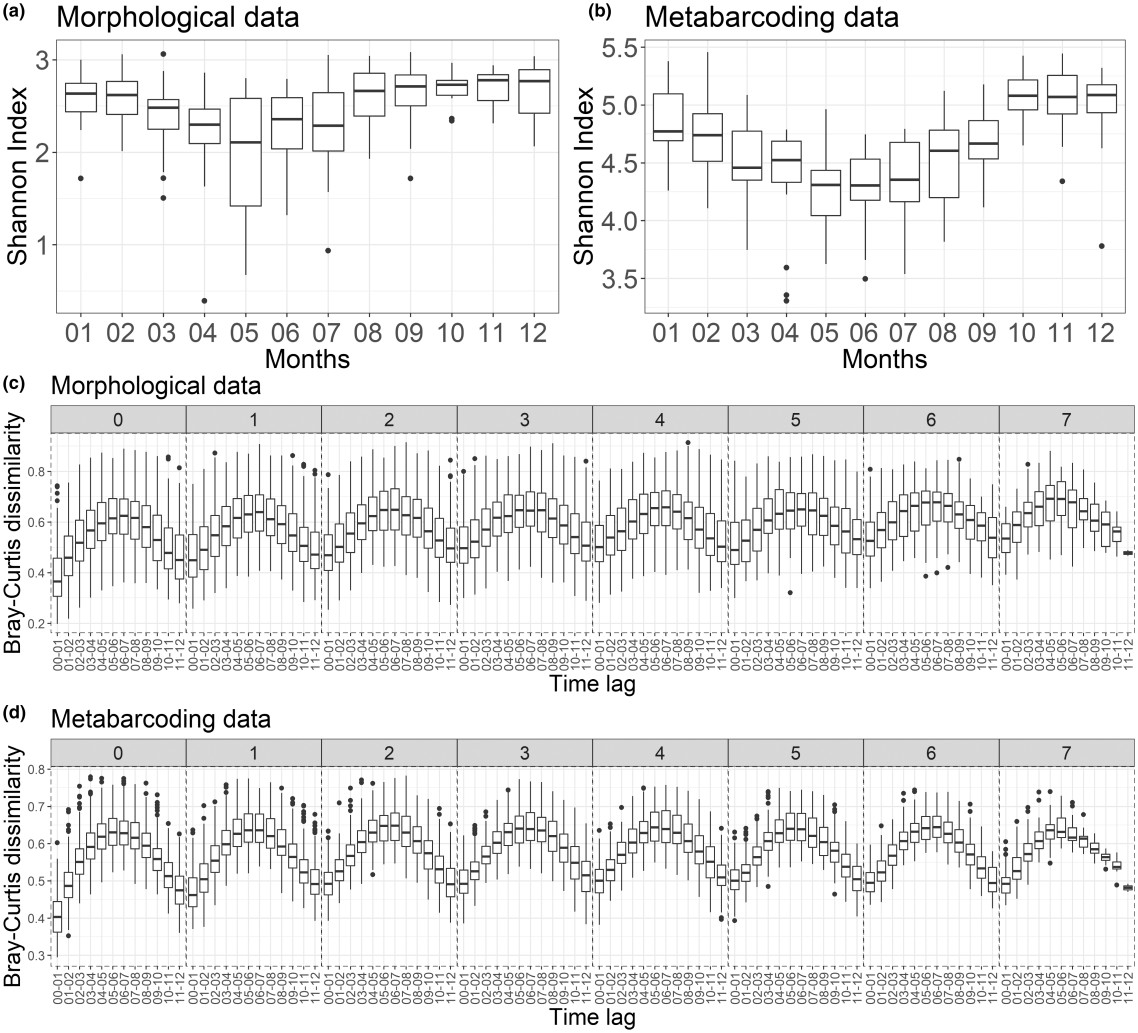
Changes in alpha and beta diversity calculated for the protist assemblages over the period 2009–2016 at the SOMLIT‐Astan sampling station. (a,b) Seasonal variations in the Shannon indexes calculated for the period 2009–2016. (c,d) Interannual recurrence of protist communities shown by the variations in the bray–Curtis dissimilarity index between samples collected along the 2009–2016 period, as a function of increasing lag between sampling dates. The lag values between samples, for each box plot correspond to a number of years (facet labels, from 0 to 7) plus a number of months (x‐axis of each facet, expressed as ranges). For example, the lag between samples considered for the first box plot is 0 years and 0 to 1 months and the lag between samples considered for the last box‐plot in 7 years and 11 to 12 months. Panels (a) and (c) are based on the morphological data set (cell counts) while graphs (b) and (d) are based on the metabarcoding data set

The protist community structure also showed clear seasonal patterns according to changes in alpha and beta diversity calculated from our morphological (mostly phytoplankton cells >10 μm) and metabarcoding (all protist 18S rDNA OTUs, size fraction >3 μm) data sets (Figure 3). Minimal Shannon diversity was recorded in spring and summer, when Chl‐*a* biomass was the highest, and maximum values were recorded in winter (Figure 3a,b). This seasonal pattern was observed for most groups although variations were encountered in the exact timing of the monthly minima of some of the phyla or classes distinguished using metabarcoding (Figure S3). For groups such as the Cercozoa, an opposite signal was recorded (Figure S3), with relatively high Shannon's diversity values in spring and summer and low values in winter. Taxa such as the MOCH‐4 (marine Ochrophytes without cultured representatives), Perkinsea or Raphidophyceae were recorded almost exclusively during winter (Figure S3).

The variations in the Jaccard and Bray‐Curtis dissimilarities ‐ calculated based upon the morphological and the metabarcoding data sets along temporal distances between samples ‐ not only confirmed the strong seasonality in the structure of the community, but also suggested gradual replacements of taxa along the year and recurrence in the annual sequence of taxa over 8 years (Figure 3b,d). The rates of changes in these similarities also showed clear temporal variations for both data sets and appeared to follow a biannual rhythm, with relative minima in February–March and October, and maxima in May–July and December–January (Figure S4). A higher variability was recorded for the morphological data set, with a decrease in similarity over time.

### 3.2 Annual succession of protist in coastal mixed environments

**FIGURE 4:**
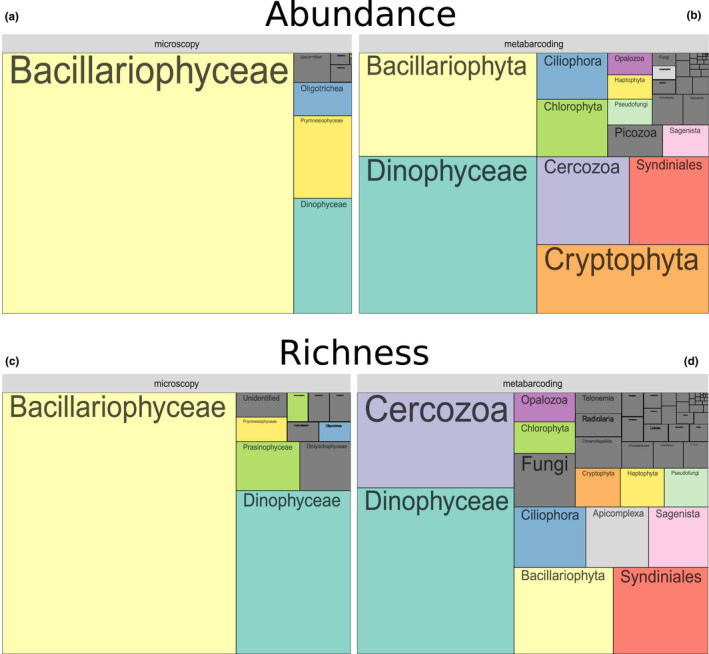
Low‐taxonomic resolution contribution of protists at the SOMLIT‐Astan time‐series station over the period 2009–2016. The tree maps show the overall contributions of the main phyla or classes to abundance of (a) the 12 main phytoplankton classes for the morphological data set; and (b) the 52 main phyla—or classes—Calculated from the metabarcoding data set and to the (c) total species or (d) OTU richness

Based on morphology (microscopy counts of cells >10 μm), diatoms were clearly the dominating group all year round and over the study period (86.5% and 74.4% of all cell counts and taxonomic entities distinguished, respectively Figure 4a,c). Dinoflagellates covered another 7.1% of all cells enumerated and accounted for 15.7% of total taxa richness. Ciliates and Haptophytes (more precisely Oligotrichea and Prymnesiophyta) accounted for 2.4% and 2.1% of all cell counts. The other groups such as Undetermined_sp., Raphidophyceae, Dictyochophyceae, Euglenophyceae, Pyramimonadophyceae, Xanthophyceae, Prasinophyceae, Undetermined_Chlorophyta, accounted each for 1% or less than 1% (Figure 4a). Each of these groups accounted for <3% of the total number of morphological entities (Figure 4c). Clear seasonal variations were encountered at phylum or class levels for absolute cell abundances (Figure S5a). The abundances of diatom cells (>10 μm) generally peaked in late spring and summer, while dinoflagellates maximal abundances were observed in late summer. Important inter‐annual variations were recorded in both the timing and intensity of the annual peaks, however. For the Prymnesiophyceae, the interannual variations were especially high, with exceptional developments of Haptophytes (corresponding to *Phaeocystis globosa* blooms) in spring 2012.

Metabarcoding uncovered a much wider diversity spectrum. Taxonomic assignation of all OTUs revealed the prevalence of Dinophyceae and diatoms in terms of relative abundance over the whole study period (29.6% and 22.1% of all reads, Figures 4b, S5b). Cryptophyta, Chlorophyta and Haptophyta that are primarily photosynthetic phyla accounted for 11.3%, 4.4% and 1.1% of all reads counts, while the heterotrophic Cercozoa, Syndiniales and Ciliophora, made up 8.1%, 7.5% and 3.5% of all read counts, respectively (Figure 4b). The contributions of the other eukaryotes, including Picozoa, Sagenista, Pseudofungi, Opalozoa, Choanoflagellida, and Telonemia, were lower (<2% of total reads, Figure 4b). In terms of OTU richness, the picture was slightly different since Dinophyceae and Cercozoa appeared as the first and second most diverse groups (18.6% and 16.6% of all OTUs, Figure 4d), followed by diatoms, Syndiniales and Ciliophora (11.4%, 10.3% and 5.8% of all OTUs). OTU richness from Sagenista (bicoecea and labyrinthulids), Opalozoa, Haptophyta, Cryptophyta, Chlorophyta, Apicomplexa, Choanoflagellida, Fungi and Telonemia ranged from 3.9% (Sagenista) to 2.1% (Telonemia) of the total number of OTUs. Other less diverse taxa belonging to 53 classes (e.g., Pseudofungi, Chrysophyceae, Picozoa, Dictyochophyceae, Bolidophyceae, Centroheliozoa, Radiolaria; see Figure 4b) accounted for less than 2% of all OTUs (Figure 4d).

Clear seasonal variations were encountered at phylum or class levels for absolute cell abundances (Figure S5a). The abundances of diatom cells (>10 μm) generally peaked in late spring and summer, while dinoflagellates maximal abundances were observed in late summer. Important inter‐annual variations were recorded in both the timing and intensity of the annual peaks, however. For the Prymnesiophyceae, the interannual variations were especially high, with exceptional developments of haptophytes (corresponding to *Phaeocystis globosa* blooms) in spring 2012. Seasonal and interannual variations were also observed when contributions to total DNA reads abundances were examined, with maximal contributions of diatoms and Dinophyceae in spring and summer, respectively, and of Cryptophyta and Chlorophyta in summer and autumn, respectively. The contribution of Cercozoa and Syndiniales (and other primarily heterotrophic, parasitic or saprotrophic groups such as the Ciliophora, Picozoa, Opalozoa and Sagenista) started to increase in early winter and were high during the first months of the year (Figure S5b).

**FIGURE 5:**
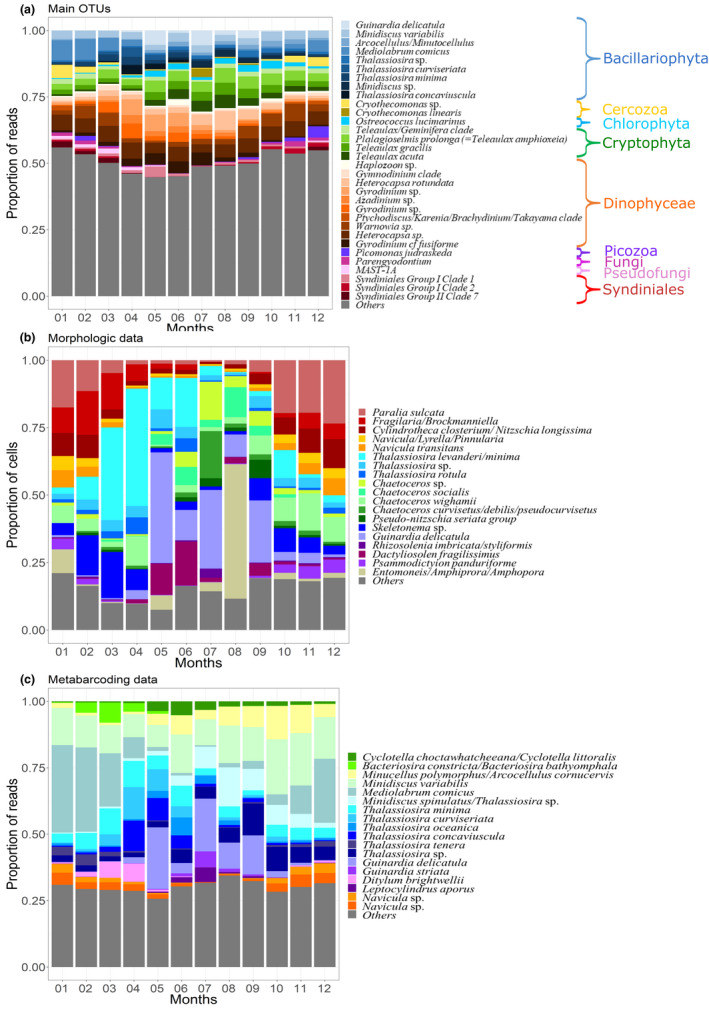
Typical seasonal variations of the dominant OTUs and overall contribution of the major diatoms species to the protist assemblage at the SOMLIT‐Astan sampling station over the period 2009–2016. The histograms show the contributions (a) to total DNA reads abundance of the 32 dominating OTUs (accounting for 51.5% of all reads), (b) of the main diatoms to total diatoms abundances (microscopy count of plankton >10 μm) and (c) of the main diatoms to total diatom reads abundances. All microscopy counts and OTUs were assigned at the highest taxonomic level. Species selected were the 10 most abundant (5 for diatoms) for at least one month, taking into account mean monthly abundances

Using the metabarcoding data set, we extracted a list of 32 OTUs that we identified as dominant based on their mean monthly contribution to total reads counts (see material and methods). These OTUs contributed to 51.5% of all reads over the study period (Figure 5a), and included diatoms, Dinophyceae, Cryptophyta, Cercozoa, Syndiniales, as well as a Chlorophyta, a Picozoa, a MAST (uncultured marine stramenopiles) and a Fungi. Sequences of both photosynthetic armoured (*Heterocapsa*) and heterotrophic naked (e.g., *Warnowia* and *Gyrodinium*) dinoflagellates dominated the sequences pools all year round. The nanoplanktonic Cryptophytes *Teleaulax amphioxeia* (*= Plagioselmis prolonga*), *T. gracilis* and *T. acuta* (all described as photosynthetic) and the green picoplanktonic algae *Ostreococcus lucimarinus* also appeared as dominant taxa. The sequences of several parasitic taxa such as the cercozoan *Cryothecomonas*, the dinoflagellates *Haplozoon* and Syndiniales, and the fungi *Parengyodontium* also showed high prevalence (Figure 5a). Diatom OTUs identified as dominating the protist communities were assigned to *Mediolabrus comicus*, *Minidiscus variabilis* and *Guinardia delicatula*, and to the genera *Thalassiosira* and *Arcocellulus* or *Minutocellus*. Although rather consistent over the 8 years, the temporal sequence showed important interannual variations (Figure S6): for example, the relative contribution of reads assigned to the parasitic *Cryothecomonas* sp. and *C. linearis* were particularly prominent during the winters 2012 and 2013, and in July 2013 and 2015, respectively (Figure S6a). Reads assigned to *Picozoa judraskeda* appeared only in 2016.

Given their prominence in both the microscopic and metabarcoding data sets, we examined in more details the seasonal dynamics of diatoms (Figure 5b,c). A list of 19 taxa and 18 OTUs identified as dominant based on mean monthly contribution to total abundances accounted for >75% and 70% of all counts/reads, respectively. In microscopic counts, autumn and winter assemblages were clearly dominated by species or genera with benthic affinities such as *Paralia* sp., *Fragilaria*/*Brockmaniella* and *Cylindrotheca closterium* /*Nitzschia longissima* (Figure 5b). These taxa were replaced, from mid‐winter to early spring, by colonial genera with pelagic affinities and in particular by *Thalassiosira* spp. (with *Thalassiosira levanderi/minima* reaching mean abundances of ~534 cells/l [35.83% of counts] in April) and *Skeletonema* spp. followed by *Dactyliosolen fragilissimus* all along spring. The dominant species in late spring and summer was *Guinardia delicatula* with the highest mean monthly abundances recorded in May and July (with ~530 cells/l for both months, 43 and 26.16% of diatom counts, respectively). The contribution of the genus *Chaetoceros* was significant from spring until early winter (with *C. curvisetus/debilis/pseudocurvisetus and C. wighamii* showing relative high contributions in July and in winter, respectively). This picture of the mean yearly sequence of diatoms appeared rather resilient over the period 2009–2016, but interannual variations were apparent, with exceptional blooms of *Skeletonema* in early spring in 2011, 2013 and 2014, and *Chaetoceros socialis* in July 2014. The contribution of the benthic diatoms associated to the genera *Entomoneis/Amphiprora/Amphora* was exceptionally high in 2011.

The analysis of the genetic data set confirmed the prevalence of the genera *Thalassiosira* and *Guinardia* during spring and summer and the relative higher contribution of *Navicula* species in winter, but gave a different picture of the seasonal succession within diatoms since the metabarcoding approach allowed deciphering the annual sequence of a pool of persistently dominant nanodiatom taxa, such as the genera *Minidiscus*, *Cyclotella*, *Arcocellulus/Minutocellus* or the species *Thalassisira minima* (Figure 5c). In winter, *Mediolabrus comicus* appeared as the dominant species while from April, and all along the summer and autumn, the contribution of *Thalassiosira* spp., *Cyclotella* and *Arcocellulus/Minutocellus* increased sequentially. If the prevalence of nanodiatoms was systematically observed every year, interannual variations in the contribution of individual species were detected (Figure S6). For example, the contribution of *Minutocellus/Arcocellulus* was particularly high during the autumn 2012 and particularly low during the autumns 2009, 2010 or 2014.

### 3.3 Ecological drivers of the temporal structure in protist community

**FIGURE 6:**
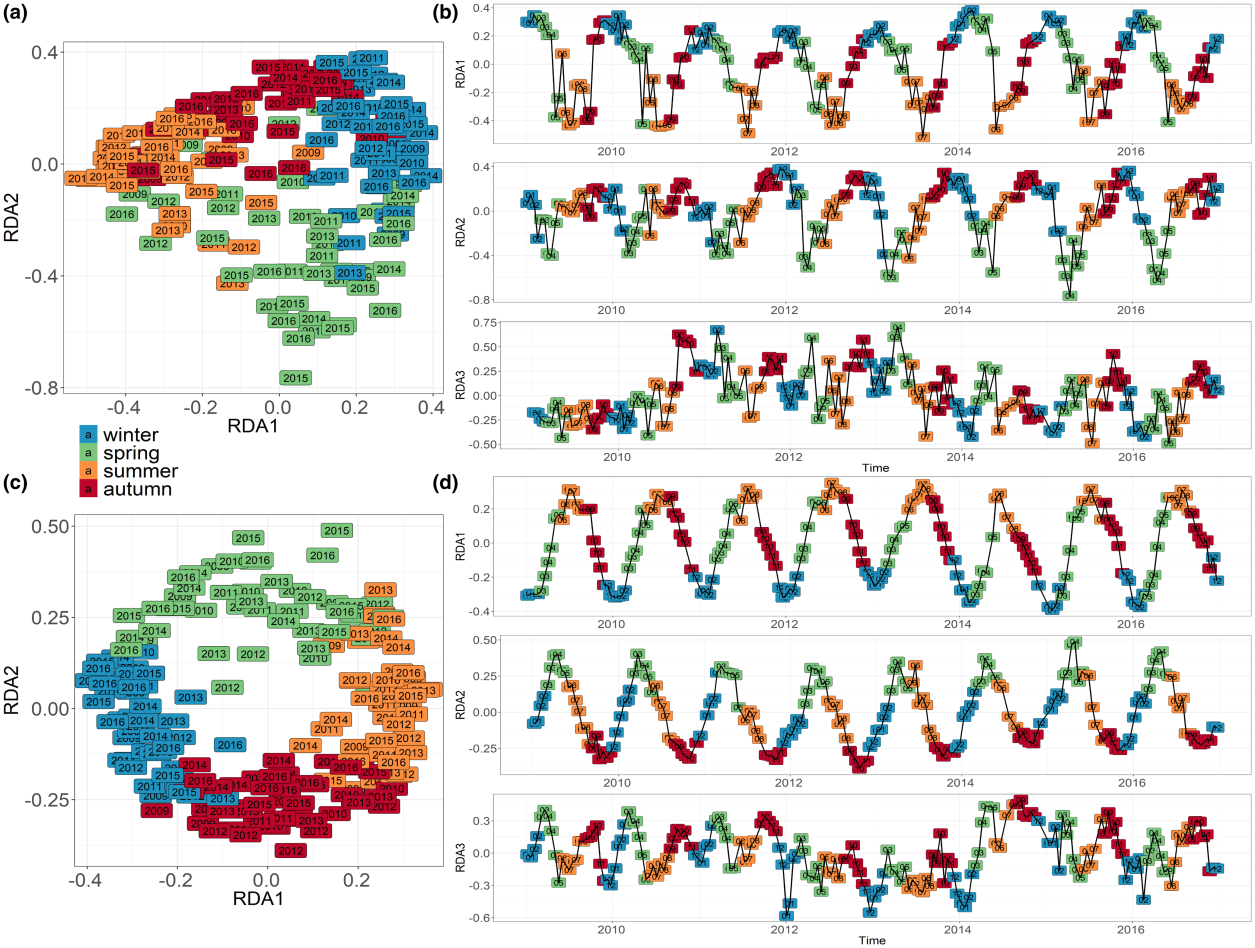
Similarity of protist communities (RDA analysis) in monthly samples over the period 2009–2016 at SOMLIT‐Astan sampling station for morphological microscopy (a,b), and DNA metabarcoding (c,d) data sets. (a,c) Annual cycle of protist communities obtained by ordination of the monthly samples through a redundancy analysis (RDA) explaining (a) 48.9% and (b) 52.2% of the total variance of the community, respectively. (b,d) Decomposition of RDA axes that reveals seasonal pattern (RDA1; 19.8%–17.8% and RDA2; 11.5%–9.3%) and biannual broadscale oscillation (RDA3; 4.8%–3.9%)

**FIGURE 7:**
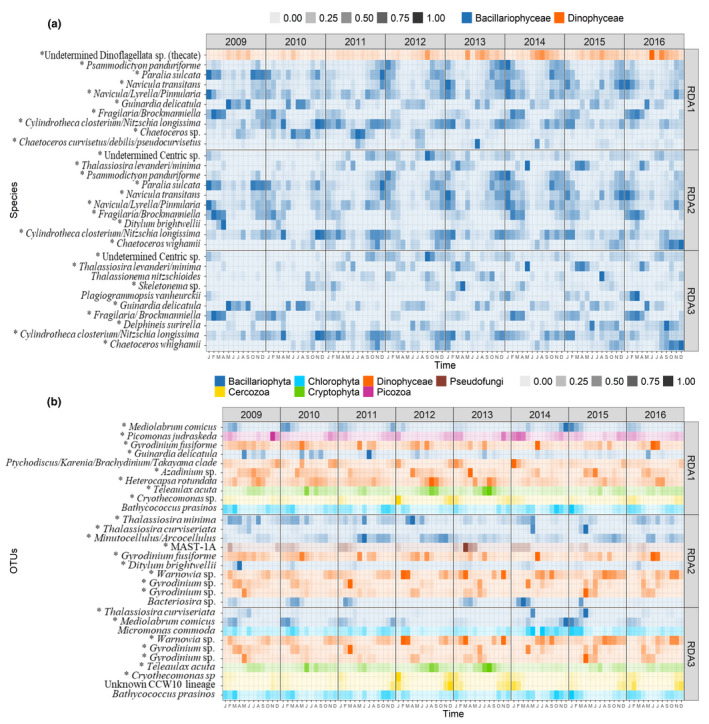
Monthly mean abundance (2009–2016), at the SOMLIT‐Astan sampling site, for (a) the morphological species and (b) molecular OTUs as a function of the first three RDA axes (see Figure 6). For each RDA axis the (a) 10 species and (b) 10 OTUs with the highest score were selected. The * indicates dominant OTUs (reported in Figure 5)

The use of a dbMEM analysis to decompose the temporal patterns of the community allowed us to detect and investigate the environmental and biological processes involved in the control of protist assemblages' dynamics at different timescales (Figure 6). Among the generated positive and negative dbMEM eigenfunctions (*n* = 55 and *n* = 129, respectively), only 52 positive dbMEM were retained after forward selection for the metabarcoding data set and 47 for the morphological data set and used as explanatory variables for a redundancy analysis (RDA; Ter Braak, 1994). These dbMEM eigenfunctions explained 48.9% of the species and 52.2% of the OTUs variability in community composition, respectively. As expected, seasonality ‐ expressed in the first two constrained axes of the RDA—explained most of the observed temporal variability (RDA1: 19.8%–17.8% and RDA2: 11.5% and 9.3%, for morphological and metabarcoding data sets, respectively; Figure 6b,d). For both data sets, the winter and summer assemblages on the one hand, and the autumn and spring assemblages on the other, were clearly distinguished on axes 1 and 2. Spring assemblages showed more interannual variability, especially when the morphological data set was considered (Figure 6a). The annual cycle was better delineated when the metabarcoding data set was considered (Figure 6c). For both data sets, the taxa/OTUs with the highest RDA1 and RDA2 scores corresponded to dominating species (Section 3.3 and Figure 5) and displayed clear seasonal variations in terms of cells or reads abundances (See Table 1 and Figure 7a,b). For the morphological data sets, the pelagic chain forming *Guinardia delicatula* and *Thalassiosira levenderi/minima* and the benthic or tychopelagic taxa *Fragilaria/Brockmanniella*, *Paralia sulcata* and *Psammodictyon pandutiforme* had the highest scores for RDA1 and/or RDA1. For the metabarcoding data set, the OTUs with the highest RDA1 and 2 scores also included *G. delicatula*, but pointed as well to nanoplanktonic diatoms (such as *Mediolabrus comicus*), and to species belonging to other phyla or classes such as the Dinophyceae and Cercozoa, all displaying strong seasonality (Figure 7b and Table 1).

**TABLE 1:** Species and OTUs driving the seasonal oscillation showed in the RDA axes 1 and 2 and the biannual broadscale oscillations observed in axes 3

	Contributi on to variation axis 1 (%)	Contributi on to variation axis 2 (%)	Contributi on to variation axis 3 (%)	Contributi on to total abundance (%)
***Morphological data set***				
*Chaetoceros curvisetus/ debilis/pseudocurvisetus*	2.4	–	–	1.3
*Chaetoceros* sp.	3.0	–	–	2.7
*Chaetoceros wighamii*		3.7	18.3	3.0
*Cylindrotheca closterium / Nitzschia longissima*	3.9	2.0	2.0	5.9
*Delphineis surirella*	–	–	0.8	1.6
*Ditylum brightwellii*	–	1.6	–	0.5
*Fragilaria/Brockmanniella*	20.2	8.5	10.5	5.7
*Guinardia delicatula*	28.2	–	8.48	8.7
*Navicula / Lyrella / Pinnularia*	2.5	1.33	–	2.7
*Navicula transitans*	2.4	3.0	–	2.7
*Paralia sulcata*	12.5	15.7	–	10.6
*Plagiogrammopsis vanheurckii*	–	–	0.97	0.4
*Psammodictyon panduriforme*	1.9	4.69	–	1.8
*Skeletonema* sp.	–	–	43.2	4.0
*Thalassiosira levanderi/minima*	–	45.7	5.0	6.6
*Thalassionema nitzschioides*	–	–	0.91	0.7
*Undetermined Centric*	–	2.5	1.2	1.9
*Undetermined Dinoflagellata (thecate)*	2.9	–	–	6.2
***Metabarcoding data set***				
*Bacterosira* sp.	–	1.9	–	0.3
*Bathycoccus prasinos*	1.5	–	1.86	0.9
Unknown CCW10 lineage	–	–	2.3	0.3
*Cryothecomonas*sp.	3.2	–	6.4	1.4
*Teleaulax acuta*	2.4	–	2.2	2.2
*Heterocapsa rotundata*	2.1	–	–	2.4
*Gyrodinium* sp.	–	5.7	2.9	2.2
*Azadinium* sp.	2.2	–	–	1.1
*Gyrodinium* sp.	–	7.1	2.5	1.7
*Ptychodiscus/Karenia/Brachydinium/Takayama clade*	1.4	–	–	0.5
*Warnowia* sp.	–	2.2	7.6	3.2
*Ditylum brightwellii*	–	2.2	–	0.4
*Guinardia delicatula*	4.1	–	–	1.5
*Gyrodinium* cf *fusiforme*	1.8	2.2	–	2.1
MAST–1A	–	2.3	–	0.5
*Micromonas commoda*	–	–	1.9	0.8
*Picomonas judraskeda*	1.5	–	–	0.7
*Mediolabrus comicus*	10.0	–	6.1	2.7
*Minutocellus/Arcocellulus*	–	2.2	–	1.0
*Thalassiosira minima*	–	2.0	–	1.2
*Thalassiosira curviseriata*	–	3.0	2.7	0.7

*Notes*: The 10 species/OTUs with the highest scores in each relative axes of the RDA were selected. The scores and the relative contributions to total abundance of the resulting list of species/OTUs are shown. For metabarcoding data, see Materials and methods section and Table S1 for assignation details.

**TABLE 2:** Parameters, abbreviations, units of measurement and origin with respective references

Environmental parameter	Abbreviation	Sample unit	Program	Reference
Temperature	T	°C	SOMLIT	https://www.somlit.fr/parametres‐et‐protocoles/
Salinity	S	–	SOMLIT	https://www.somlit.fr/parametres‐et‐protocoles/
pH	pH	–	Calculated	Gac et al. (2020)
Air‐sea CO₂ flux	FCO₂	mmol C m^−2^ d^−1^	Calculated	Gac et al. (2020)
Oxygen	O	ml/l	SOMLIT	https://www.somlit.fr/parametres‐et‐protocoles/
Ammonium	$\mathrm{NH}₄{}^{+}$	μM	SOMLIT	https://www.somlit.fr/parametres‐et‐protocoles/
Nitrite	${\mathrm{NO}}^{-}₂$	μM	SOMLIT	https://www.somlit.fr/parametres‐et‐protocoles/
Nitrate	${\mathrm{NO}}^{-}₃$	μM	SOMLIT	https://www.somlit.fr/parametres‐et‐protocoles/
Dissolved silicates	SiOH₄	μM	SOMLIT	https://www.somlit.fr/parametres‐et‐protocoles/
Phosphate	$\mathrm{PO}₄{}^{3-}$	μM	SOMLIT	https://www.somlit.fr/parametres‐et‐protocoles/
Particulate organic carbon	POC	μg/l	SOMLIT	https://www.somlit.fr/parametres‐et‐protocoles/
Particulate organic nitrate	PON	μg/l	SOMLIT	https://www.somlit.fr/parametres‐et‐protocoles/
Suspended matter	MES	mg/l	SOMLIT	https://www.somlit.fr/parametres‐et‐protocoles/
Chlorophyll‐*a*	Chl‐*a*	μg/l	SOMLIT	https://www.somlit.fr/parametres‐et‐protocoles/
NOP Isotopes ratio	Delta 15 N	‰	SOMLIT	https://www.somlit.fr/parametres‐et‐protocoles/
Photosinthetic available radiation	PAR	E m^−2^ d^−1^	NASA	https://modis.gsfc.nasa.gov/data/dataprod/par.php
Coefficient of light attenuation	Kd_490	m^−1^	NASA	https://modis.gsfc.nasa.gov/data/dataprod/kd_490.php
Tide amplitude	–	m	SHOM	https://data.shom.fr/
Rainfall height	–	mm	Meteo France	https://meteofrance.com/
Wind speed	–	m/s	Meteo France	https://meteofrance.com/
North Atlantic Oscillation Index	NAO	–	NOAA	https://www.ncdc.noaa.gov/teleconnections/nao/

Axis 3 of the RDA (4.8% and 3.9% of the variance explained for the morphological and metabarcoding data sets, respectively, Figure 6b,d) expressed broad scale oscillations and a persistent biannual rhythm in the protist community dynamics. In the morphological data set, *Skeletonema* sp. contributed most to axis 3 of the RDA. *G. delicatula* and *Chaetoceros wighamii* also showed high contribution. In the metabarcoding data set, the winter diatom *M. comicus* and the Cercozoan *Cryothecomonas*, that exhibit a parasitic lifestyles, had the highest contribution to this axis.

**FIGURE 8:**
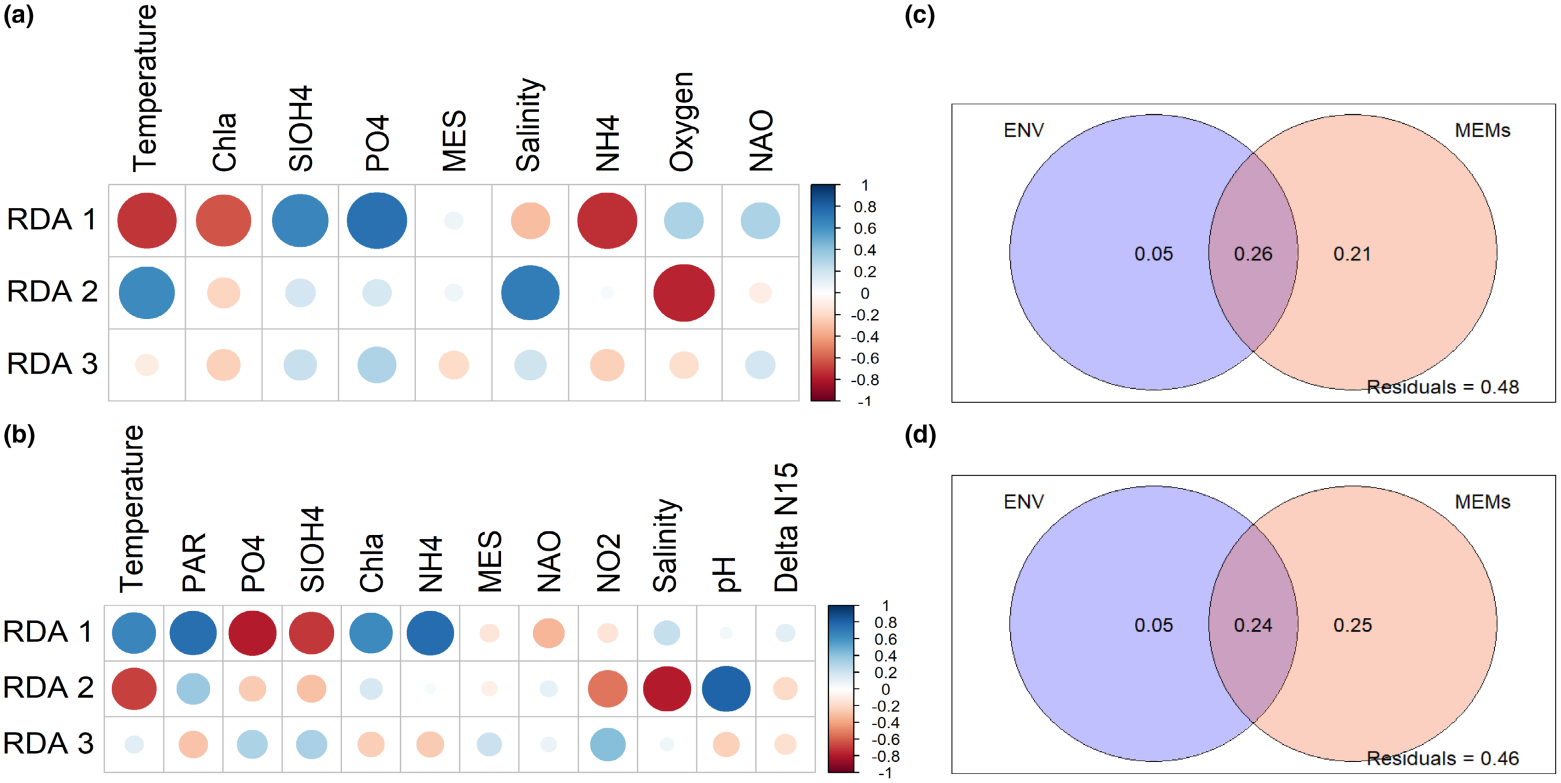
Spearman's correlation calculated between the environmental variables and the RDA axes (a,c), and variance partitioning analyses between environmental drivers and dbMEM (b,d). Spearman's correlations were computed between each axes of the RDA and each environmental parameter selected for (a) morphology and (b) metabarcoding. Variance partitioning between selected environmental variables and dbMEM was also calculated for (c) morphology and (d) metabarcoding data, respectively

To investigate the environmental factors that primarily drive seasonal protist assemblages, we calculated Spearman's rank correlation coefficients between the potential explanatory variables and the first three axes of the RDA (Figure 8a,b). Here, we considered the environmental variables selected by forward selection, respectively, for the morphological and metabarcoding data sets. Temperature, phosphates (${{\text{PO}}_{4}}^{3-}$), silicates (${{\text{SiO}}_{4}}^{-}$), ammonia (${{\text{NH}}_{4}}^{+}$), Chl‐*a*, salinity, suspended matter (MES) and the North Atlantic Oscillation (NAO) Index were selected for both data sets (Figure 8a,b). Oxygen was selected only for microscopy (Figure 8a); and PAR, nitrate (${{\text{NO}}_{2}}^{-}$), pH, and Delta N15 only for metabarcoding (Figure 8b). The analyses suggested that macronutrients (${{\text{PO}}_{4}}^{3-}$, ${{\text{NH}}_{4}}^{+}$ and to a less extent ${{\text{SiO}}_{4}}^{-}$), together with temperature, PAR, and chlorophyll‐*a* that showed the highest correlations with RDA1 were important drivers of plankton seasonal successions. Temperature, salinity, oxygen, pH and ${{\text{NO}}_{2}}^{-}$ showed the highest correlations with RDA2 (Figure 8a,b). Even though environmental variables alone only accounted for 5% of the variance, a large part of the variations in the community was explained by the temporal structure of environmental factors (26 and 24% for both data sets, respectively, Figure 8c,d). Overall, the temporal organization of the community explained most of the variations when considered together with the temporal structure of the environment (47% and 49%).

## 4 DISCUSSION

### 4.1 Resilient cyclic successions of protists in coastal pelagic habitats

Using morphological and DNA metabarcoding approaches, we clearly identified annual succession patterns of taxa in the Western English Channel over the period 2009–2016. The cyclic pattern was more distinct using metabarcodes (see Figure 6a vs c), compared to microscopic counts (146 morphological taxa). Moreover, the genetic sequences enabled to capture more phyla and to reach a much finer taxonomic resolution (15,271 OTUs belonging to 53 different phyla and classes) and gave access to the dynamics of nanoplanktonic taxa that are dominant at the site. For example, the nanoplanktonic diatoms *Minidiscus variabilis* and *Mediolabrus comicus*, are known major players of the microbial communities in the Western English Channel (Arsenieff et al., 2020; Foulon et al., 2008; Not et al., 2004). Although the samples were collected onto 3 μm filters, the set of sequences obtained included sequences of picoplanktonic taxa such as the green algae *Ostreococcus lucimarinus*, *Bathycoccus prasinos* and *Micromonas* spp. also prevalent in the coastal waters off Roscoff (Foulon et al., 2008; Not et al., 2004). DNA metabarcoding could also capture the dynamics of naked dinoflagellates taxa (*Gyrodinium* and *Gymnodinium* species) and heterotrophic, parasitic or endosymbiotic microeukaryotes such as the MAST that are bacterivorous protists (Massana et al., 2006), *Cryothecomonas*, and Syndiniales species. Taxa such as *Cryothecomonas* that infects diatoms and especially the genus *Guinardia* (Drebes et al., 1996; Peacock et al., 2014), and the Syndiniales that parasite dinoflagellates (Chambouvet et al., 2008) are involved in the control of phytoplankton blooms and thus in the overall stability of the system.

To our knowledge, this study is the first to show such regularities and stability in the planktonic community composition in a permanently‐mixed pelagic habitat. In the WEC along the Brittany coasts, the hydrodynamics, mostly driven by intense tidal currents and salinity gradients, is at the origin of strong physical and biogeochemical heterogeneity. This results in a mosaic of interconnected benthic and pelagic habitats, with, for example, frequent changes of sediment types and associated benthic communities along the nearshore and offshore gradient (Cabioch et al., 1968; Dauvin, 2008; Delavenne et al., 2013; Gac et al., 2020). By transporting species from and to adjacent habitats, tidal currents are known to increase dispersal, which is an important process in structuring communities (Vellend et al., 2010). In habitats influenced by tidal mixing, high contributions of benthic protists to the water column communities are classically observed (Forster et al., 2016, Hernandez Farinas et al., 2017); this phenomenon is amplified in winter, when pelagic species are less abundant and winds increase vertical mixing (Mann & Lazier, 1991). Nevertheless, our results demonstrate that the induced high rates of emigration and immigration do not disrupt the seasonal oscillations in diversity which appears as a rather common feature of marine microbial communities (see Fuhrman et al., 2015 for bacteria and Lambert et al., 2019 or Giner et al., 2019 for protists; Figure 6). By increasing the diversity and enhancing bentho‐pelagic coupling (and potentially the interactions between species), these forces may on the contrary favour the overall stability of the system (Cardinale et al., 2012). The idea that biodiversity buffers ecosystem changes against environmental variations (Tilman, 1999; Tilman et al., 2006; Loreau & de Manzancourt, 2013) matches results obtained from manipulated microbiomes (Fernandez‐Gonzalez et al., 2016) and theoretical studies (Dakos et al., 2009). It could explain the strong temporal relationship that links species richness and community‐level properties (Cottingham et al., 2001; Griffin et al., 2009; Loreau et al., 2001).

### 4.2 The annual sequence of dominant protists in temperate tidally‐mixed habitats

With observations conducted over 8 years using both microscopy and DNA metabarcoding, our study improves our knowledge of pelagic protists in a tidally‐mixed coastal environment. Regarding phototrophic organisms, our study confirmed the importance of diatoms (by far the most numerous taxa >10 μm enumerated under microscopy), dinoflagellates and green algae, but also highlighted the importance of Cryptophyta. The DNA metabarcoding analysis also provided new data about the seasonal sequence of important heterotrophic dinoflagellates (i.e., Dinophyceae and Syndiniales) harbouring diverse trophic modes, and that of parasitic Cercozoa. Along the years 2009–2016, the prominence of the chain‐forming species *Guinardia delicatula* during spring and summer was confirmed by both the morphological and metabarcoding data sets. This species is emblematic of the spring and summer diatoms bloom in the Roscoff area (Grall, 1972; Martin‐Jezequel, 1983; Sournia et al., 1987; Guilloux et al., 2013; Arsenieff et al., 2019). It is more generally a very common species in plankton samples of the English Channel and North Sea (Caracciolo et al., 2021; Widdicombe et al., 2010) and it appears to be particularly successful in temperate tidally‐mixed habitats (Gomez & Souissi, 2007; Wiltshire et al., 2008; Peacock et al., 2014; Schlüter et al., 2012; Hernández‐Fariñas et al., 2014). Our analyses also tracked the classical annually repeated sequence of diatoms that involves the development of microplanktonic pelagic chain‐forming species in spring (typically *Thalassiosira* spp., *G. delicatula*, *Chaetoceros* spp.), as well as benthic and tychopelagic species in winter (e.g., *Paralia* sp. and *Navicula* spp.). An annual sequence of nanodiatoms (involving species of the genera *Minidiscus*, *Mediolarus, Thalassiosira* and *Arcocellulus / Minutocellus*) was specifically revealed by the metabarcoding approach. The prevalence of nanodiatoms, and especially of *Minidiscus/Mediolabrus* spp. at the SOMLIT‐Astan station has been confirmed since species of these genera have been isolated in culture using serial dilution from samples collected at different seasons at the SOMLIT‐Astan station (Arsenieff et al., 2020). Nanodiatoms have been identified as prominent members of diatoms assemblages in other marine systems when adequate detection techniques (cultures, electron microscopy or HTS) were implemented (Leblanc et al., 2018; Ribera d'Alcalà et al., 2004; Percopo et al., 2011).

Microphytoplanktonic dinoflagellates are usually present at relatively low abundances in species microscopic counts in tidally‐mixed waters off Roscoff (Sournia et al., 1987; Guilloux et al., 2013). However, contribution of dinoflagellates reads in the molecular data set was high all year round according to this study. Sequences corresponding to the dominant reads were mostly assigned to nanoplanktonic species or naked species. Two OTUs assigned to the genus *Heterocapsa* including the thecate species *H. rotundata* dominated read counts for the whole period. This ubiquitous mixotrophic dinoflagellate, that has the potential to switch from phototrophy to partial heterotrophy (Millette et al., 2017), may be favoured at our tidally‐mixed coastal site, especially in August when light starts decreasing. Interestingly, *H. rotundata* was also identified as a dominant taxon in the adjacent Penzé estuary (Chambouvet et al., 2008), and as most abundant in recent microscopic counts obtained from our time‐series station where nanoplanktonic dinoflagellates were targeted (data not shown). Some other dominant dinoflagellate OTUs detected in our metabarcoding data set are either heterotrophic or potentially mixotrophic (*Gyrodinium*, *Gymnodinium*, *Azadinium*, *Warnowia* etc.), and some of them are purely parasitic (Syndiniales). The naked dinoflagellates *Gyrodinium* and *Gymnodinium* spp. were also identified as prominent members of the phytoplankton community in the stratified waters of the WEC, off Plymouth (Widdicombe et al., 2010) and showed an increasing trend in abundance after 2001 (Hernández‐Fariñas et al., 2014). These dinoflagellates, that seem to thrive all year round, may be key predators for diatoms. The increasing trend in average abundance of some dinoflagellates and the decrease in diatoms has been recently documented in the Central North Atlantic Ocean and in the North Sea (Leterme et al., 2005; Zhai et al., 2013), as well as in the English Channel (Widdicombe et al., 2010).

Cryptophyta are important members of protists communities in coastal waters. Their prominence in different regions of the ocean has been revealed using microscopy (Jochem, 1990), but also via flow cytometry, since the phototrophic members of this group can be distinguished based on its phycoerythrin fluorescence (Li & Dickie 2001). Recent DNA metabarcoding analyses have also revealed their prominence in coastal waters at Helgoland Roads, North Sea (Käse et al., 2020). At the SOMLIT‐Astan station, sequences identical to different species of the genus *Teleaulax* were abundant in read counts. The highest proportion of Cryptophyta reads were assigned to *Plagioselmis prolonga* (=*Teleaulax amphioxeia*), a phototrophic species with a bentho‐pelagic life‐cycle (Altenburger et al., 2020) involved in complex symbioses with the ciliate *Mesodinium rubrum* (Qiu et al., 2016). In addition, the later species has an interesting behaviour consisting of periodic dispersion away from the strong superficial tidal currents, thus minimizing flushing losses (Crawford & Purdie, 1991).

We are aware that the description of the typical seasonal sequence of protists species provided herein is still incomplete. Both microscopy and metabarcoding can provide biased data, since the former does not consider the smallest taxa while the latter which was applied to cells collected onto >3 μm filters probably underestimated the contribution of picoplanktonic species since those cells probably pass through the filter). In our study, the contribution of Haptophyta was probably underestimated since most species in this group are nano‐ or picoplanktonic and were thus not reported in our morphological data set. Also, the primers used for metabarcoding do not perfectly match with all eukaryotic sequences especially in groups such as the Haptophyta or Dinoflagellata (Egge et al., 2015; Balzano et al., 2015, McNichol et al., 2021). Moreover, metabarcoding can overestimate or underestimate the proportions of taxa for which DNA is more easily extracted and amplified (Santi et al., 2021). Likewise, an overestimation of the contribution to sequences reads obtained from natural samples of groups, such as the dinoflagellates, that harbour very high numbers of 18S rRNA gene copies, is a commonly reported bias (Gong & Marchetti, 2019). However, the two data sets we used are complementary and allowed us to add important information about the dynamics of dominant protists thriving in permanently mixed waters of the Western English Channel. A deeper analysis of species dynamics in the different phyla for the metabarcoding data set will certainly provide more information in the future, especially since reference sequences databases and taxonomic frameworks (required for accurate assignations to genus or species levels) are constantly being updated and curated (Guilloux et al., 2013; Berney et al., 2017; Glöckner et al., 2017; del Campo et al., 2018).

### 4.3 Environmental versus community intrinsic drivers of protistan plankton seasonal dynamics

According to our analyses, a large amount of variation (almost 50%) in the protist community structure at SOMLIT‐Astan depends on temporal effects. About half of this temporal effect is accounted for by temporally structured environment variations, especially in PAR, temperature, salinity and macronutrients (notably ${{\text{PO}}_{4}}^{3}$, ${{\text{SiO}}_{4}}^{2-}$, ${{\text{NH}}_{4}}^{+}$, Figure 8a,b). The metabarcoding data set also indicate the importance of pH and ${{\text{NO}}_{2}}^{-}$ and to a lesser extend to Delta N15 which depend on the sources of nitrate.

The fact that time alone, that is, temporal structures generated by the species assemblage itself (creating autocorrelation) contributes >20% to the variance of the community (Figure 8c) suggests that intrinsic biological factors (i.e., species interactions, reproductive dynamics, and/or self‐regulation of species development; in other words, self‐organization properties of the whole biological community; Odum, 1988; Picoche & Barraquand, 2019) are also critical, and significantly contribute to pacing the plankton community. Microscopic organisms are indeed known to be involved in complex and dynamic networks of interactions (i.e., grazing, parasitism, mutualism, quorum sensing, etc; Kivi et al., 1993; Dakos et al., 2009; Platt et al., 2010; Bjorbækmo et al., 2020) that are tightly regulating the dynamics of individual species within the whole community structure. Recent analyses of plankton dynamics in the WEC at L4 station are supporting the hypothesis that, beyond extrinsic forcing by the environment, predator–prey interactions play a role in influencing temporal changes in plankton populations (Barton et al., 2020).

Bi‐annual variations in the protist community dynamics were also identified from analyses of both data set (Figure S4 and Figure 6). In some ecosystems, rhythmic depletions of resources appear to be at the origin of bimodality (and multimodality) in phytoplankton dynamics (Mellard et al., 2019); however, in our tidally‐mixed coastal station, nutrients are never completely depleted (Figure 2). The number of benthic species detected as prominent in surface waters (in particular among diatoms, Figure 5) suggests a tight coupling between benthic and pelagic compartments in the English Channel, which is strengthened in winter, when tidal mixing or winds provoke the resuspension of sediments in the water column. However, in our dbMEM analysis, neither tidal amplitude nor wind appeared as a major influential parameter. This yearly bimodality could then be caused by intrinsic plankton biological factors such as endogenous rhythmicity or interactions between species. To better decipher how intrinsic biotic interactions could drive the dynamics of these communities, modelling approaches that take into account biotic interactions (e.g., Picoche & Barraquand, 2019) should be explored, integrating the whole taxonomic and functional spectrum that coexist in space and time, including viruses, prokaryotes, and metazoans.

## 5 CONCLUSIONS AND PERSPECTIVE

This study describes the seasonal dynamics of protist communities in a coastal permanently‐mixed pelagic habitat. Our study points to relative resilience of this diverse community over the 8 year period studied. However, in environments such as the coastal waters of the EC that support one of the busiest shipping lanes in the world, important fishing ports, and an increasing demographic pressure, these seasonal cycles may be particularly vulnerable to the combined effects of natural climate variability and local anthropogenic pressures (Dauvin, 2008; Tréguer et al., 2014; Gac et al., 2020; Siano et al., 2021). In this context, monitoring activities involving both classical microscopy and metagenomics approaches, such as those conducted along the EC coasts (Breton et al., 2000; Widdicombe et al., 2010; Hernandez‐Farinas et al., 2017; Kenitz et al. 2017; Käse et al., 2020), should be maintained and developed in the long term. These longitudinal surveys are critical to track and predict future changes that may disrupt the overall resilience of the system, in order to ultimately identify and deploy protective measures to guarantee all the services that these systems provide to the society (Cardinale et al., 2012).

## AUTHOR CONTRIBUTIONS

Nathalie Simon, Fabrice Not, Nicolas Henry and Mariarita Caracciolo designed the study. Fabienne Rigaut‐Jalabert, Thierry Cariou and the crew of the Neomysis sampled onboard. Fabienne Rigaut‐Jalabert and Loïc Guilloux produced the taxa counts. Mark Hoebeke helped with the construction and maintenance of the phytoplankton counts databases. Fabienne Rigaut‐Jalabert, Samuel Forsans and Nathalie Simon contributed to the corrections and validation of the taxonomic counts data set. Fabienne Rigaut‐Jalabert and Sarah Romac produced the genetic data. Thierry Cariou produced the hydrological data and Thierry Cariou and Yann Bozec contributed to the validation of the hydrological data validation. Jean‐Philippe Gac produced the final estimations of pH and FCO_2_. Eric Goberville provided the map. Samuel Chaffron helped with the calculations of the PAR data. Mariarita Caracciolo and Nicolas Henry analysed the data and produced the scripts and final figures. Mariarita Caracciolo, Nicolas Henry, Eric Thiébaut, Fabienne Rigaut‐Jalabert, Sarah Romac and Nathalie Simon wrote the manuscript. All authors contributed to the discussions that led to the final manuscript, revised it and approved the final version.

## CONFLICT OF INTEREST

All authors declare that they have no conflict of interest.

### OPEN RESEARCH BADGES

This article has earned an Open Data, for making publicly available the digitally‐shareable data necessary to reproduce the reported results. The data is available at Raw environmental data: https://meteofrance.com/, https://www.somlit.fr/en/, https://www.somlit.fr/en/, https://data.shom.fr/, https://modis.gsfc.nasa.gov/data/dataprod/, and https://coastwatch.pfeg.noaa.gov/. Microscopic counts file: https://doi.org/10.5281/zenodo.5033180. Metabarcoding raw reads: EuropeanNucleotide Archive (ENA): PRJEB48571. rDNA 18S V4 OTU table: https://doi.org/10.5281/zenodo.5032451.

## Supporting information


**FIGURE S1** Summary of analyses performed on the OTU contingency table. Each white box represents a manipulation of the data necessary to arrive at the final result in the color box.
**FIGURE S2** Mean yearly variations recorded for the hydrological and meteorological parameters at the SOMLIT‐Astan time‐series station in the period 2009–2016. All measurements were obtained for high neap tides periods. PAR8day is the photosynthetically available radiation calculated as the average light received during the 8 days that preceded each sampling dates. Kd490 is intended as the diffuse attenuation coefficient for downwelling irradiance at 490 nm (for more details about each parameter see Material & Methods section).
**FIGURE S3** Monthly variations of the Shannon Index. For metabarcoding, alpha diversity was calculated at the class level or phylum level. The absence of data during some months for certain classes is linked to the absence of that OTUs during those months.
**FIGURE S4** Monthly variations in the ecosystem turn‐over at the SOMLIT‐Astan station for the period 2009–2016 as estimated from the protist community: (a) Bray‐Curtis dissimilarities, (b) Jaccard distances as calculated from metabarcoding data and (c) monthly means euclidian distances as calculated from environmental data
**FIGURE S5** Monthly variations in the cell abundance (a) and contribution to reads abundances (b) of dominating high‐rank taxonomic groups at the SOMLIT‐Astan time‐series station over the period 2009–2016
**FIGURE S6** Temporal variations in the monthly contributions of dominating protists or diatoms at the SOMLIT‐Astan time‐series station over the period 2009–2016. (a) The contributions to total DNA reads abundance of the dominating OTUs; (b) contributions of the main diatoms to total species abundances; (c) contribution of the main diatoms to total diatom reads abundances. OTUs/species selected were the 10 most abundant for at least one month, when mean monthly abundances were taken into account (5 most abundant for diatoms)

**Table S1** Taxonomic assignation of the 32 dominant OTUs (shaded rows) and considering only diatoms (see text for the method used) at the SOMLIT‐Astan time‐series station. The automatic assignation using the PR2 database was checked and refined by comparing the corresponding sequences to references using BLAST (ref). The SILVA assignations of these sequences was also checked for some dinoflagellate sequences. The origin of the best blast sequences listed (most of which had 100% similarity with our query sequence) and of the corresponding strains or isolates were carefully examined before taking the final taxonomic assignation decision. Cultured strains that were isolated from the SOMLIT‐Astan Station are highlighted in bold. For some of the reference sequences, taxonomic assignation was not considered valid (assignation written in gray). Microalgae Culture Collection ^1^Trefault et al. (2020), ^2^Nanjappa,D. et al. (2013), ^3^Laza‐Martinez etal. (2012)

## Data Availability

Raw environmental data: https://meteofrance.com/, https://www.somlit.fr/en/, https://www.somlit.fr/en/, https://data.shom.fr/, https://modis.gsfc.nasa.gov/data/dataprod/, and https://coastwatch.pfeg.noaa.gov/. Microscopic counts file: https://doi.org/10.5281/zenodo.5033180. Metabarcoding raw reads: European Nucleotide Archive (ENA): PRJEB48571. rDNA 18S V4 OTU table: https://doi.org/10.5281/zenodo.5032451.
