# Supplementary material for: Seasonal dynamics of marine protist communities in tidally mixed coastal waters
Source: Mol Ecol. 2022 Jun 16;31(14):3761–83. doi: 10.1111/mec.16539 (PMC9543310; doi:10.1111/mec.16539)
Supplement: Supplementary file 1 — FIGURE S1 Summary of analyses performed on the OTU contingency table. Each white box represents a manipulation of the data necessary to arrive at the final result in the color box. FIGURE S2 Mean yearly variations recorded for the hydrological and meteorological parameters at the SOMLIT‐Astan time‐series station in the period 2009–2016. All measurements were obtained for high neap tides periods. PAR8day is the photosynthetically available radiation calculated as the average light received during the 8 days that preceded each sampling dates. Kd490 is intended as the diffuse attenuation coefficient for downwelling irradiance at 490 nm (for more details about each parameter see Material & Methods section). FIGURE S3 Monthly variations of the Shannon Index. For metabarcoding, alpha diversity was calculated at the class level or phylum level. The absence of data during some months for certain classes is linked to the absence of that OTUs during those months. FIGURE S4 Monthly variations in the ecosystem turn‐over at the SOMLIT‐Astan station for the period 2009–2016 as estimated from the protist community: (a) Bray‐Curtis dissimilarities, (b) Jaccard distances as calculated from metabarcoding data and (c) monthly means euclidian distances as calculated from environmental data FIGURE S5 Monthly variations in the cell abundance (a) and contribution to reads abundances (b) of dominating high‐rank taxonomic groups at the SOMLIT‐Astan time‐series station over the period 2009–2016 FIGURE S6 Temporal variations in the monthly contributions of dominating protists or diatoms at the SOMLIT‐Astan time‐series station over the period 2009–2016. (a) The contributions to total DNA reads abundance of the dominating OTUs; (b) contributions of the main diatoms to total species abundances; (c) contribution of the main diatoms to total diatom reads abundances. OTUs/species selected were the 10 most abundant for at least one month, when mean monthly abundances were taken into account (5 most [file MEC-31-3761-s001.docx]

**Figure S1**

**Summary of analyses performed on the OTU contingency table.** Each white box represents a manipulation of the data necessary to arrive at the final result in the color box.

**
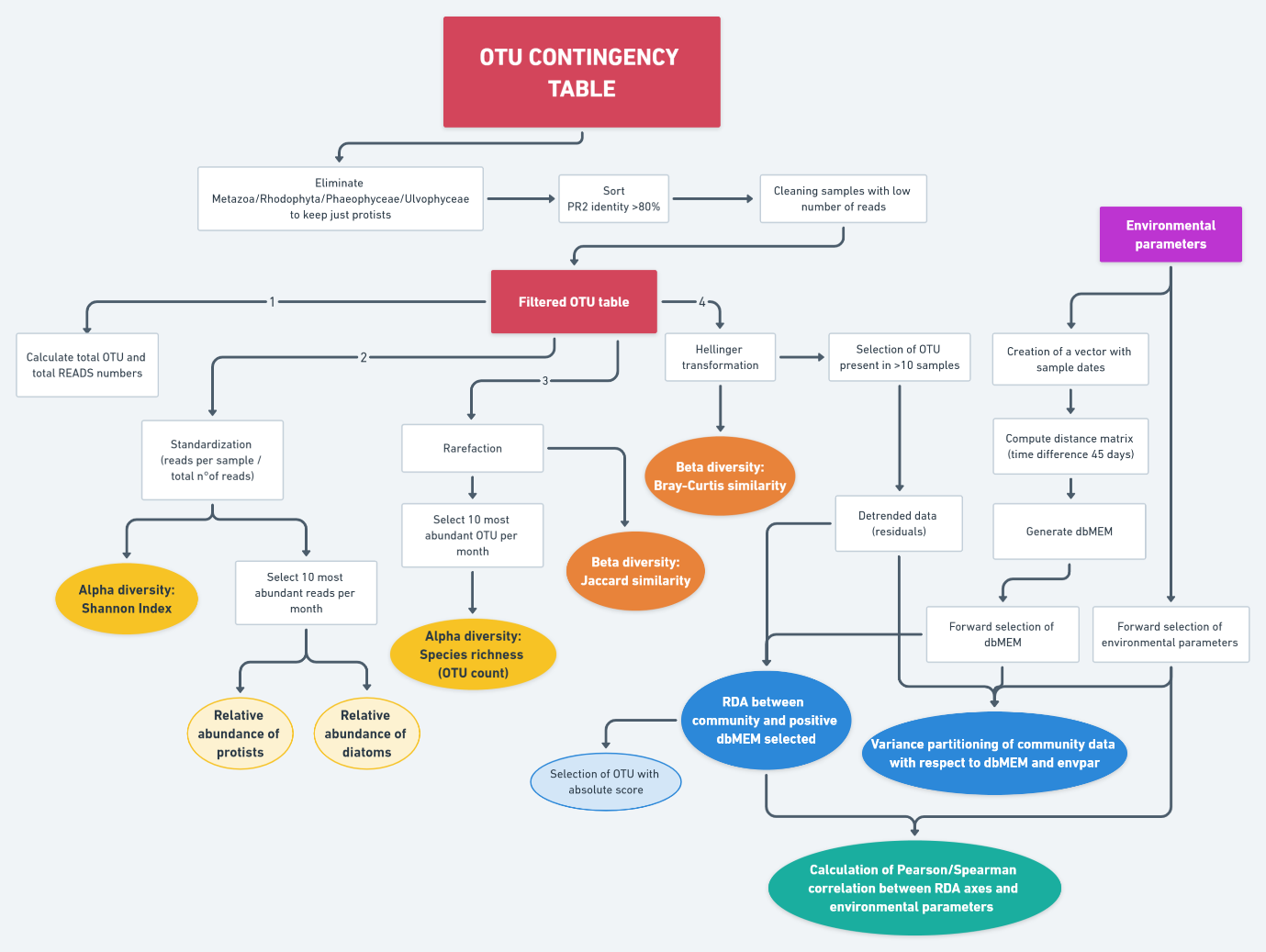
**

**Figure S2
Mean yearly variations recorded for the hydrological and meteorological parameters at the SOMLIT-Astan time-series station in the period 2009-2016.** All measurements were obtained for high neap tides periods. PAR8day is the photosynthetically available radiation calculated as the average light received during the 8 days that preceded each sampling dates. Kd490 is intended as the diffuse attenuation coefficient for downwelling irradiance at 490 nm (for more details about each parameter see Material & Methods section).

**
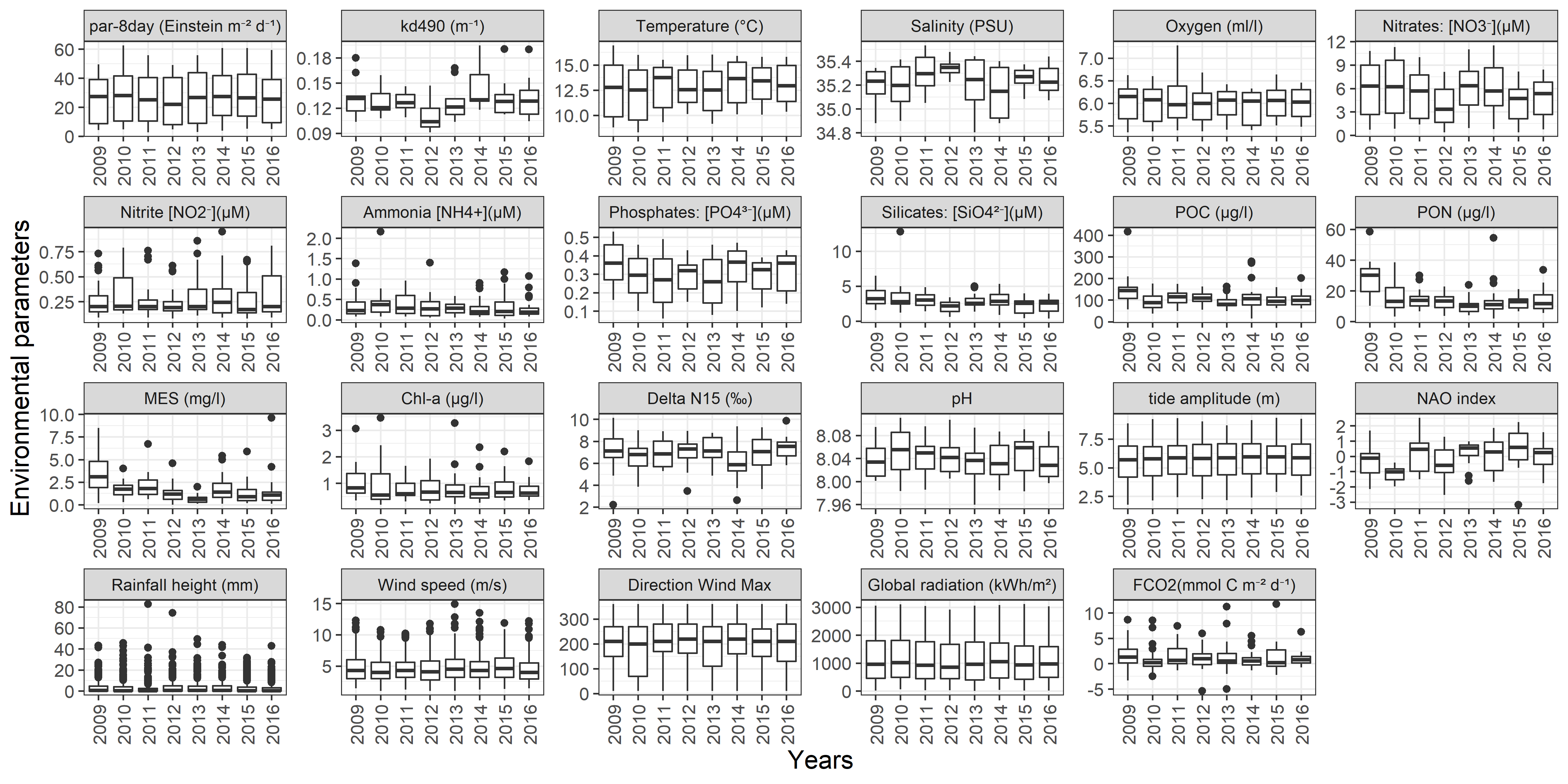
**

**Figure S3**

**Monthly variations of the Shannon Index.** For metabarcoding, alpha diversity was calculated at the class level or phylum level. The absence of data during some months for certain classes is linked to the absence of that OTUs during those months.

**
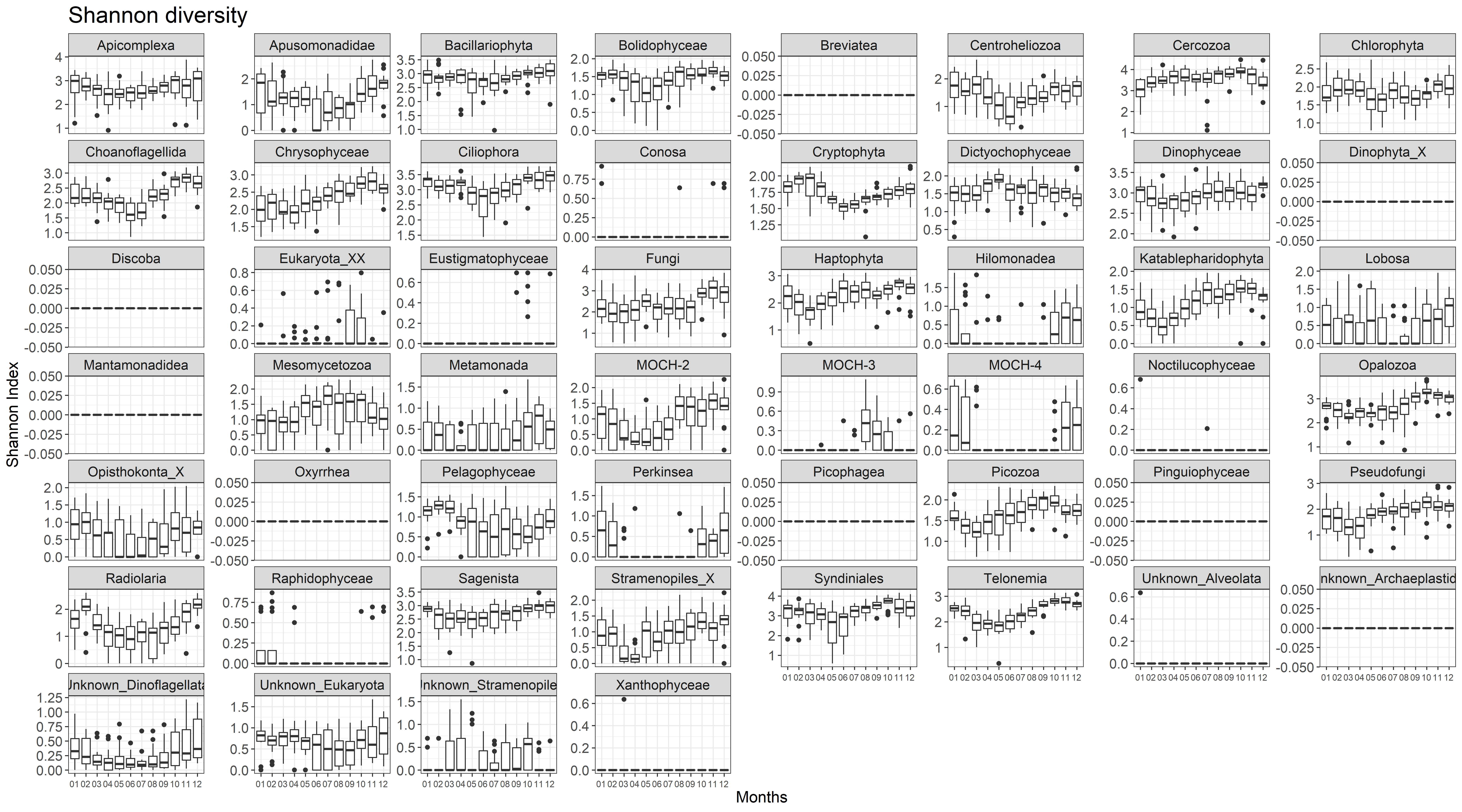
**

**Figure S4**

**Monthly variations in the ecosystem turn-over at the SOMLIT-Astan station for the period 2009-2016** as estimated from the protist community: (**A)** Bray-Curtis dissimilarities, (**B**) Jaccard distances as calculated from metabarcoding data and (**C**) monthly means euclidian distances as calculated from environmental data.

**
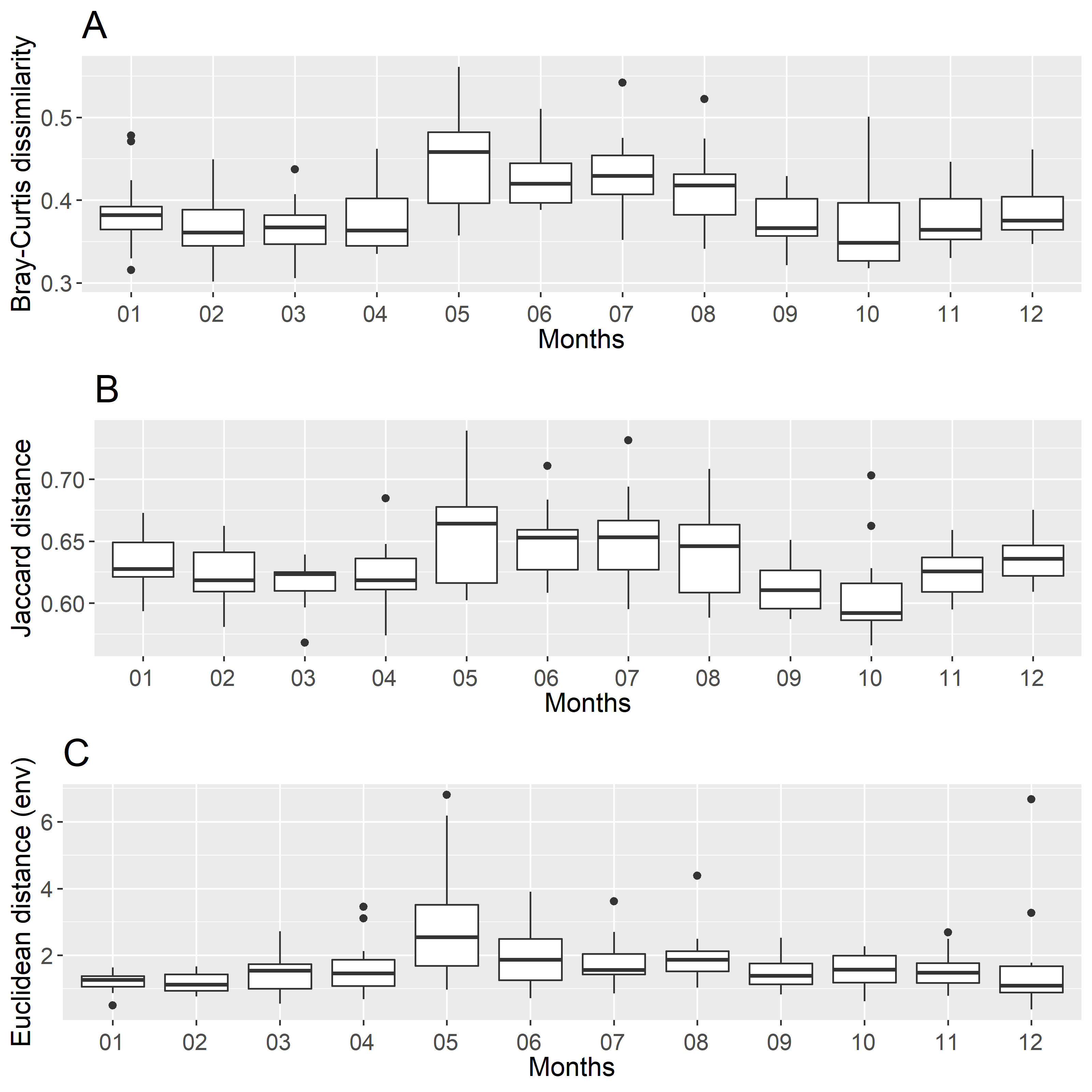
**

**Figure S5**

**Monthly variations in the cell abundance (A) and contribution to reads abundances (B) of dominating high-rank taxonomic groups at the SOMLIT-Astan time-series station over the period 2009-2016.**

**
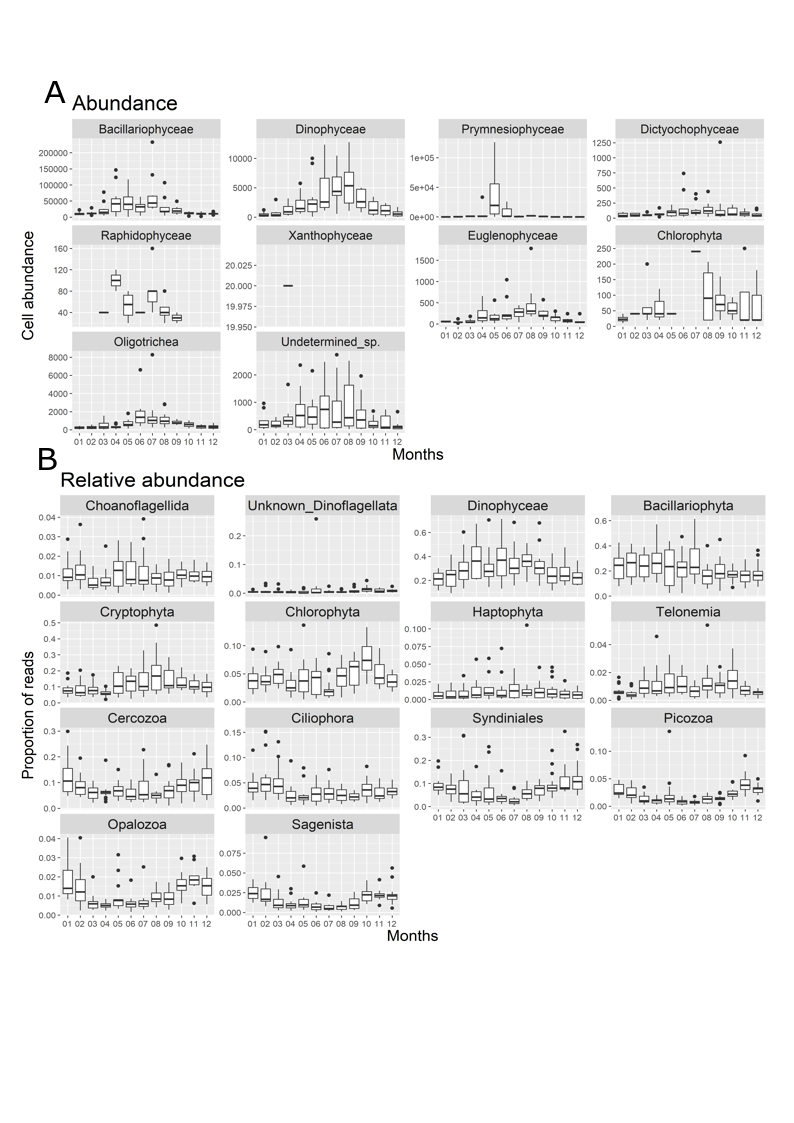
**

**Figure S6**

**Temporal variations in the monthly contributions of dominating protists or diatoms at the SOMLIT-Astan time-series station over the period 2009-2016.** (**A**) The contributions to total DNA reads abundance of the dominating OTUs; (**B**) contributions of the main diatoms to total species abundances; (**C**) contribution of the main diatoms to total diatom reads abundances. OTUs/species selected were the 10 most abundant for at least one month, when mean monthly abundances were taken into account (5 most abundant for diatoms).

**A**

**
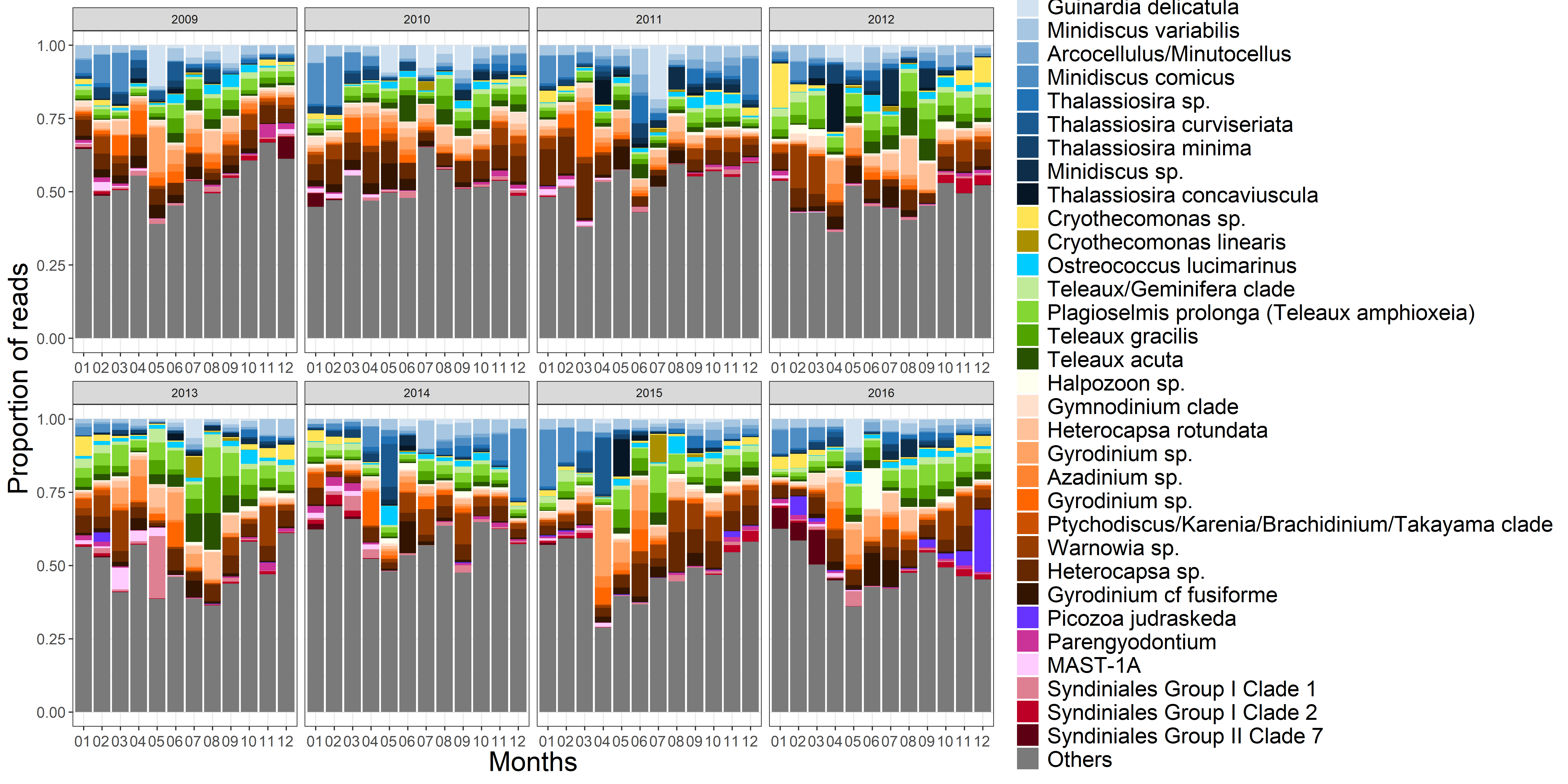
**

**B**

**
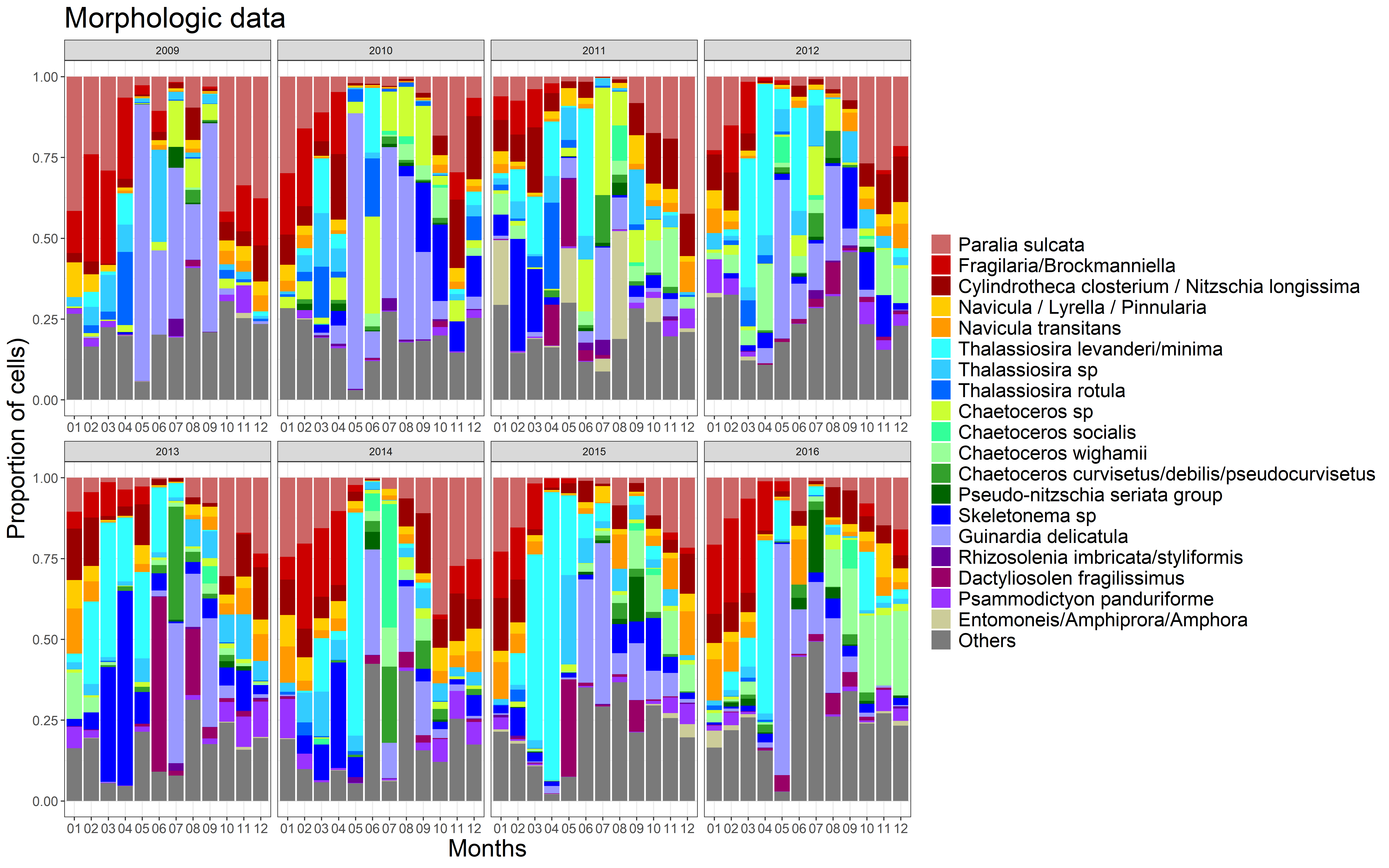
**

**C
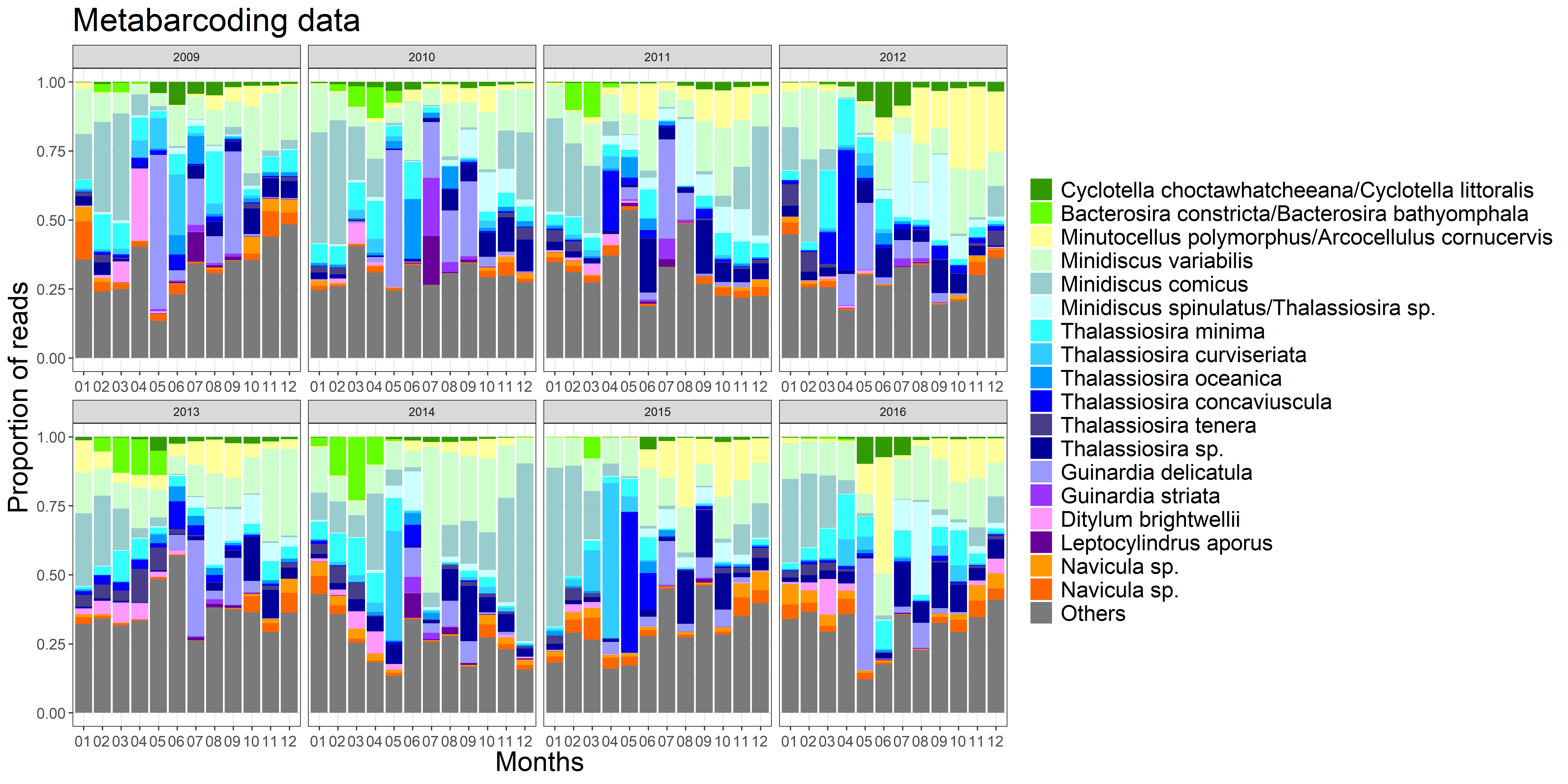
**
