# Supplementary material for: Seasonal dynamics of marine protist communities in tidally mixed coastal waters
Source: Mol Ecol. 2022 Jun 16;31(14):3761–83. doi: 10.1111/mec.16539 (PMC9543310; doi:10.1111/mec.16539)
Supplement: Supplementary file 2 — Table S1 Taxonomic assignation of the 32 dominant OTUs (shaded rows) and considering only diatoms (see text for the method used) at the SOMLIT‐Astan time‐series station. The automatic assignation using the PR2 database was checked and refined by comparing the corresponding sequences to references using BLAST (ref). The SILVA assignations of these sequences was also checked for some dinoflagellate sequences. The origin of the best blast sequences listed (most of which had 100% similarity with our query sequence) and of the corresponding strains or isolates were carefully examined before taking the final taxonomic assignation decision. Cultured strains that were isolated from the SOMLIT‐Astan Station are highlighted in bold. For some of the reference sequences, taxonomic assignation was not considered valid (assignation written in gray). Microalgae Culture Collection 1Trefault et al. (2020), 2Nanjappa,D. et al. (2013), 3Laza‐Martinez etal. (2012) [file MEC-31-3761-s002.pdf]

Table S1 : Taxonomic assignation of the 32 dominant OTUs (shaded rows) and considering only diatoms (see text for the method used) at the SOMLIT-Astan time-series station. The automatic assignation using the PR2 database was checked and refined by comparing the corresponding sequences to references using BLAST (ref). The SILVA assignations of these sequences was also checked for some dinoflagellate sequences. The origin of the best blast sequences listed (most of which had 100% similarity with our query sequence) and of the corresponding strains or isolates were carefully examined before taking the final taxonomic assignation decision. Cultured strains that were isolated from the SOMLIT-Astan Station are highlighted in bold. For some of the reference sequences, taxonomic assignation was not considered valid (assignation written in gray). Microalgae Culture Collection [1] Trefault et al. (2020), [2] Nanjappa,D. et al. (2013), [3] Laza-Martinez etal. (2012)

PR2 taxonomic group	OTU (PR2 species rank taxonomy_OTU short name_4 first letters of OTU ID code)	Manual assignation					
		Best blasts	Corresponding isolates or strains	% identity	NCBI Taxonomy	SILVA Taxonomy	Final assignation
Bacillariophyta	Guinardia_delicatula_baed	MH782132.1	**RCC5799**	100	*Guinardia delicatula*		*Guinardia delicatula*
Bacillariophyta	Minidiscus_a751	MN528627.1	**RCC4657**	100	*Minidiscus variabilis*		*Minidiscus variabilis*
Bacillariophyta	Polar-centric-Mediophyceae_92af	LC189088; JN934677.1	NIES-3970; RCC2270	100	*Minutocellus; Arcocellulus*		*Minutocellus/Arcocellulus*
Bacillariophyta	Polar-centric-Mediophyceae_dd92	MH011734	GF367-18S_12	100	*Thalassiosira*		*Thalassiosira sp.*
Bacillariophyta	Polar-centric-Mediophyceae_X_sp._38e7	MN528601	**RCC4660**	100	*Minidiscus comicus*		*Minidiscus comicus*
Bacillariophyta	Thalassiosira_c57e	JN934676	RCC2265	100	*Thalassiosira minima*		*Thalassiosira minima*
Bacillariophyta	Thalassiosira_cefd	MH843669.1; DQ514870	RCC4582; CC03-15	100	*Minidiscus sp.; Shionodiscus oestrupii var venrickae*		*Minidiscus* sp.[1]
Bacillariophyta	Thalassiosira_379f	MN528650	**RCC5154**	100	*Thalassiosira curviseriata*		*Thalassiosira curviseriata*
Bacillariophyta	Thalassiosira_concaviuscula_1b87	AJ810857	Type strain MHtc1	100	*Thalassiosira concaviuscula*		*Thalassiosira concaviuscula*

Bacillariophyta	Bacterosira_a2c6	KT692951.1; DQ514894.1; DQ514877.1	SMDCO1286; NB04-B6; CCMP991	100	*Bacterosira constricta; Bacterosira bathyomphala; Bacterosira sp.*	*Bacterosira sp.*
Bacillariophyta	Cyclotella_46d6	JQ217341.1; JQ217340.1	KMMCC B147; KMMCC B214	100	*Cyclotella choctawhatcheeana; Cyclotella litoralis*	*Cyclotella sp.*
Bacillariophyta	Ditylum_brightwellii_1e0f	AY485444.1; X85386.1	CCMP358; CCAP1022/2	100	*Ditylum brightwellii*	*Ditylum brightwellii*
Bacillariophyta	Leptocylindrus_d056	KC814810.1; AJ535175.1	SZN-B651; KM9950	100	*Leptocylindrus aporus; Leptocylindurs danicus*	*Leptocylindrus aporus*[2]
Bacillariophyta	Navicula_92d5	KT861019.1	RCC3092	100	*Navicula*	*Navicula* sp.
Bacillariophyta	Polar-centric-Mediophyceae_71e5	AJ810858.1	"type strain MHtt1"	100	*Thalassiosira tenera*	*Thalassiosira tenera*
Bacillariophyta	Radial-centric-basal-Coscinodiscophyceae_X_sp._4884	KJ671697.1; KT861015.1	"Strain 12"; RCC2966	100; 99.74	*Guinardia striata*	*Guinardia striata*
Bacillariophyta	Thalassiosira_oceanica_8902	DQ514878.1	CCMP1001	100	*Thalassiosira oceanica*	*Thalassiosira oceanica*
Cercozoa	Cryothecomonas_aestivalis_2a84	AF290541.1; AF290539.1	"Strain 2"; "Strain 1"	99.74; 99.48	*Cryothecomonas aestivalis*	*Cryothecomonas aestivalis*
Cercozoa	Cryothecomonas-lineage_7aac	KY979993.1; GQ144679.1	NY13S_83; APCC MC-1Cryo	99.74; 99.22	Cryothecomonas sp.	Cryothecomonas sp.
Chlorophyta	Ostreococcus_lucimarinus_59f6	MT117941.1	BCC118000	100	*Ostreococcus lucimarinus*	*Ostreococcus lucimarinus*
Cryptophyta	Cryptomonadales_X_39cf	MK956825.1; MK956143.1	"strain 10"; single cell isolate	100	*Teleaulax amphioxeia (=Plagioselmis prolonga)*	*Teleaulax amphioxeia (=Plagioselmis prolonga)*[3]
Cryptophyta	Cryptomonadales_X_a5a5	JQ966995.1; JQ966994.1	Cr7EHU; Cr6EHU	100	*Teleaulax gracilis*	*Teleaulax gracilis*
Cryptophyta	Cryptomonadales_X_f546	MK956814.1; HM126531.1; KY980327.1; KY980204.1	"Strain 07"; SCAP K-1486	100	*Teleaulax acuta/ Teleaulax amphioxeia*	*Teleaulax acuta*[3]
Cryptophyta	Cryptomonadales_XX_sp._7e7d	KY980182.1; MK956818.1	BH46_33; RCC5152	99,73	*Teleaulax amphioxeia; Geminifera cryophila*	*Teleaulax/Geminifera clade*

Dinoflagellata	Dinoflagellata_bcc6	KY980212.1; KY980192.1	BH46_94; BH46_57	100	*Adenoides eludens*	*Haplozoon* sp.	*Haplozoon* sp.
Dinophyceae	Dinophyceae_fcf1	KY980285.1	BH46_144	100	*Heterocapsa rotundata*	*Heterocapsa* sp.	*Heterocapsa* sp.
Dinophyceae	Dinophyceae_3301	AF274267.1; DQ388464.1	CCMP680; CCMP1542	100	*Heterocapsa rotundata*	*Heterocapsa rotundata*	*Heterocapsa rotundata*
Dinophyceae	Dinophyceae_8fd6	FN669511.1; FN669510.1	GCGMS0407NS; GDMS0704YD	99.74	*Gyrodinium cf guturula; G. dominans*	*Gyrodinium* sp.	*Gyrodinium sp.*
Dinophyceae	Dinophyceae_09a4	KY980035.1	NY13S	100	*Warnowia* sp.	*Gymnodinium clade, Gyrodiniellum*	Gymnodinium clade
Dinophyceae	Dinophyceae_XXX_sp._af41	KP790170.1; FJ947040	5 AR-2015; BSL-2009a	99.21	*Warnowia* sp.	*Warnovia* sp.	*Warnovia* sp.
Dinophyceae	Dinophyceae_XXX_sp._24ad	FJ024299.1	Clone LM83	99.74	*Gyrodinium helveticum*	*Gyrodinium* sp.	Gyrodinium sp.
Dinophyceae	Dinophyceae_XXX_sp._034f	KY980399.1	BH65_136	100	*Pentapharsodinium* sp.	*Azadinium* sp.	Azadinium sp.
Dinophyceae	Dinophyceae_XXX_sp._3d28	KU640194,1; KU314867.1; EF492506.1; HM067010.1	unnamed; unnamed; NEPCC734; MC728-D5	98.43	*Ptychodiscus noctiluca; Karlodinium veneficum; Karlodinium micrum; Takayama acrotrocha*	Gymnodiniphy-cidae	*Ptychodiscus/Karenia/Bra-chidinium/Takayama clade*
Dinophyceae	Gyrodinium_75e1	KY980394.1; KY980272.1; KY980220.1; AB120002.1	BH46_129; BH46_109; BH46_107; single cell	100	*Heterocapsa rotundata* (3 sequences); *Gyrodinium fusiforme*	*Gyrodinium* sp.	*Gyrodinium* cf *fusiforme*
Eukaryota	Eukaryota_b342	KU747081.1; KT582539.1; KM096193.1	SZ1; NIOLM_56; LF562	100	*Parengyodontium album; Halophytophtora vesicula; Engyodontium* sp.	*Parengyodon-tium*	*Parengyodontium*
Picozoa	Picozoa_XXX_18f8	JX988758.1	Unnamed culture	100	*Picomonas judraskeda*		*Picomonas judraskeda*
Pseudofungi	MAST-1A_XX_sp._0c52						MAST-1A
Syndiniales	Dino-Group-I-Clade-1_X_sp._5e71						Syndiniales GroupI Clade1

Syndiniales	Dino-Group-I-Clade-1_X_sp._ac94	Syndiniales GroupI Clade1
Syndiniales	Dino-Group-II-Clade-7_X_sp._86d3	Syndiniales GroupII Clade7
